# Structure of magnesium selenate enneahydrate, MgSeO_4_·9H_2_O, from 5 to 250 K using neutron time-of-flight Laue diffraction

**DOI:** 10.1107/S2052520615006824

**Published:** 2015-05-26

**Authors:** A. Dominic Fortes, Dario Alfè, Eduardo R. Hernández, Matthias J. Gutmann

**Affiliations:** a Department of Earth Sciences, University College London, Gower Street, London WC1E 6BT, England; b Department of Earth and Planetary Sciences, Birkbeck, University of London, Malet Street, London WC1E 7HX, England; c ISIS Facility, Rutherford Appleton Laboratory, Harwell Science and Innovation Campus, Didcot, Oxfordshire OX11 0QX, England; d Instituto de Ciencia de Materiales de Madrid (ICMM-CSIC), Campus de Cantoblanco, 28049 Madrid, Spain

**Keywords:** magnesium selenate enneahydrate, dodecamer, neutron diffraction

## Abstract

The complete structure of MgSeO_4_·9H_2_O has been refined from neutron single-crystal diffraction data obtained at 5, 100, 175 and 250 K. It is monoclinic, space group *P*2_1_/*c*, *Z* = 4, with unit-cell parameters *a* = 7.222 (2), *b* = 10.484 (3), *c* = 17.327 (4) Å, β = 109.57 (2)°, and *V* = 1236.1 (6) Å^3^ [ρ_calc_ = 1770 (1) kg m^−3^] at 5 K. The structure consists of isolated [Mg(H_2_O)_6_]^2+^ octahedra, [SeO_4_]^2−^ tetrahedra and three interstitial lattice water molecules, all on sites of symmetry 1. The positions of the H atoms agree well with those inferred on the basis of geometrical considerations in the prior X-ray powder diffraction structure determination: no evidence of orientational disorder of the water molecules is apparent in the temperature range studied. Six of the nine water molecules are hydrogen bonded to one another to form a unique centrosymmetric dodecamer, (H_2_O)_12_. Raman spectra have been acquired in the range 170–4000 cm^−1^ at 259 and 78 K; *ab initio* calculations, using density functional theory, have been carried out in order to aid in the analysis of the Raman spectrum as well as providing additional insights into the geometry and thermodynamics of the hydrogen bonds. Complementary information concerning the thermal expansion, crystal morphology and the solubility are also presented.

## Abstract 2

Single-crystal neutron diffraction, *ab initio* calculations and Raman spectroscopy are applied to study the structure and hydrogen bonding of magnesium selenate enneahydrate, a recently discovered new hydration state in *M* ^2+^ *X*O_4_ hydrates and a unique example of a material with a cation: (oxy)anion:water ratio of 1:1:9, giving novel insights into the polymerization of water in highly hydrated materials.

## 1. Introduction   

Crystalline hydrates with the general formula *M* ^2+^ *X*O_4_·*n*H_2_O are very well known for *n* in the range 1–7 with *X* = S, Se, Cr and Mo, and *M* ^2+^ being any of a wide range of first-row transition metals, as well as Mg^2+^ and Cd^2+^, although not all integer values of *n* occur for a given cation or oxyanion composition. Until quite recently, there was a conspicuous gap for *n* = 8–10, and just one known example with *n* = 11, being MgSO_4_·11H_2_O (aka meridianiite). Motivated principally by an interest in ‘planetary’ cryohydrates (Fortes & Choukroun, 2010 ▶), a series of systematic studies have been carried out in an effort to understand the apparently limited occurrence of these materials with *n* ≥ 8. This work has revealed new examples of 11-hydrates that are isotypic with the sulfate compound, MgCrO_4_·11H_2_O (Fortes & Wood, 2012 ▶; Fortes *et al.*, 2013 ▶) and MgSeO_4_·11H_2_O (Fortes, 2015 ▶), and shown that hydrates with *n* = 8 and 9 can be synthesized (Fortes *et al.*, 2012*a* ▶,*b* ▶; Fortes, 2014 ▶). All of the *M* ^2+^ sulfate octa- and enneahydrates^1^ prepared thus far are metastable, being crystallized only by rapidly quenching small droplets of aqueous solution in liquid nitrogen, after which they typically undergo solid-state transformations over periods of minutes to hours into other stable hydrates. However, this proved not to be the case for magnesium selenate, where it was determined that MgSeO_4_·11H_2_O undergoes incongruent melting to form MgSeO_4_·9H_2_O apparently in equilibrium with aqueous solution, and furthermore that MgSeO_4_·7H_2_O crystals stored in a freezer formed pseudomorphs after the heptahydrate composed of the enneahydrate (Fortes, 2015 ▶).

The solid–liquid phase equilibria in the MgSeO_4_–H_2_O binary system have been a source of some disagreement, there being significant differences between the observations of Meyer & Aulich (1928 ▶) and those of Klein (1940 ▶). In neither instance was the behaviour below the freezing point of ice characterized, the solubility curves and eutectic being found by extrapolation: the highest stable hydrate found in this system, and the phase believed to be in equilibrium at the eutectic (266 K), was MgSeO_4_·7H_2_O, the structure of which was recently determined (Fortes & Gutmann, 2014 ▶). Nevertheless, attempts to grow single crystals of magnesium selenate enneahydrate by evaporation of an aqueous solution in air at 269 K were successful and this allowed us to carry out a more thorough characterization of the material’s properties and to obtain more accurate and complete structural parameters, as reported here.

## 2. Experimental   

### 2.1. Sample preparation   

An aqueous solution of magnesium selenate was prepared as follows: commercially available aqueous H_2_SeO_4_ (Sigma–Aldrich 481513, 40 wt %) was diluted to 25 wt % H_2_SeO_4_ (1.72 M) with distilled water (Alfa–Aesar, ACS Reagent Grade, 36645), which was then heated to ∼ 340 K. To this liquid was added a molar excess of powdered MgO (Sigma Aldrich 342793, > 99% trace metals basis, −325 mesh). Since this synthesis, unlike the reaction with basic magnesium carbonate, is quiescent its progress was followed with a Tecpel hand-held pH meter. Once the pH of the solution stabilized at 8.80, the supernatant liquid was decanted, triply filtered and left to stand. Evaporation in air at room temperature resulted in the precipitation of cm-sized crystals of MgSeO_4_·6H_2_O. After a further round of recrystallization from distilled water the phase purity of the hexahydrate was verified by X-ray powder diffraction (see §S1 of the supplementary information).

Crystals of MgSeO_4_·6H_2_O were crushed to a coarse powder and dissolved in distilled water to a concentration of ∼ 35 wt % MgSeO_4_, the liquid then being left to evaporate in a petri dish in a refrigerated workshop (air temperature = 269 K). Crystals up to 1 cm in length, having a morphology distinct from either the heptahydrate (acicular prisms with a square cross section) or the hexahydrate (pseudo-hexagonal plates, Fig. S1), grew from solution over a period of a few days. One of these was extracted from solution, dried and powdered under liquid nitrogen before being subjected to X-ray powder diffraction analysis using a custom-made Peltier cold stage (Wood *et al.*, 2012 ▶) mounted on a PANAlytical X’Pert Pro powder diffractometer. The X-ray powder diffraction pattern of this material was identical to that reported previously for MgSeO_4_·9H_2_O obtained by quenching droplets of the solution in liquid nitrogen (Fortes, 2015 ▶).

Subsequent attempts to form single crystals produced only MgSeO_4_·7H_2_O. However, these crystals transformed to MgSeO_4_·9H_2_O (as pseudomorphs after the heptahydrate) during 11 days storage in a freezer at 253 K. This material was used to seed the growth of new 9-hydrate single crystals from solution. Two cycles of recrystallization using this seed material yielded good quality single crystals up to 5 mm in length, which were harvested for analysis.

**Figure 1:**
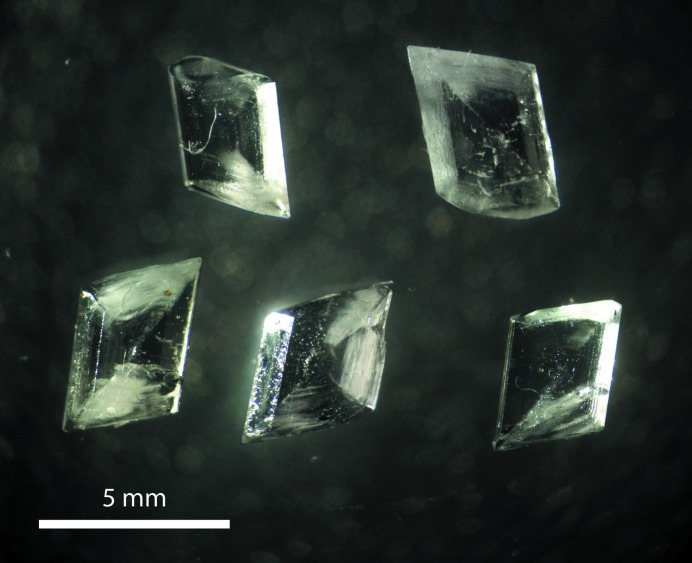
Microphotograph of MgSeO_4_·9H_2_O crystals in air illustrating their representative platy rhomboidal habit.

**Figure 2:**
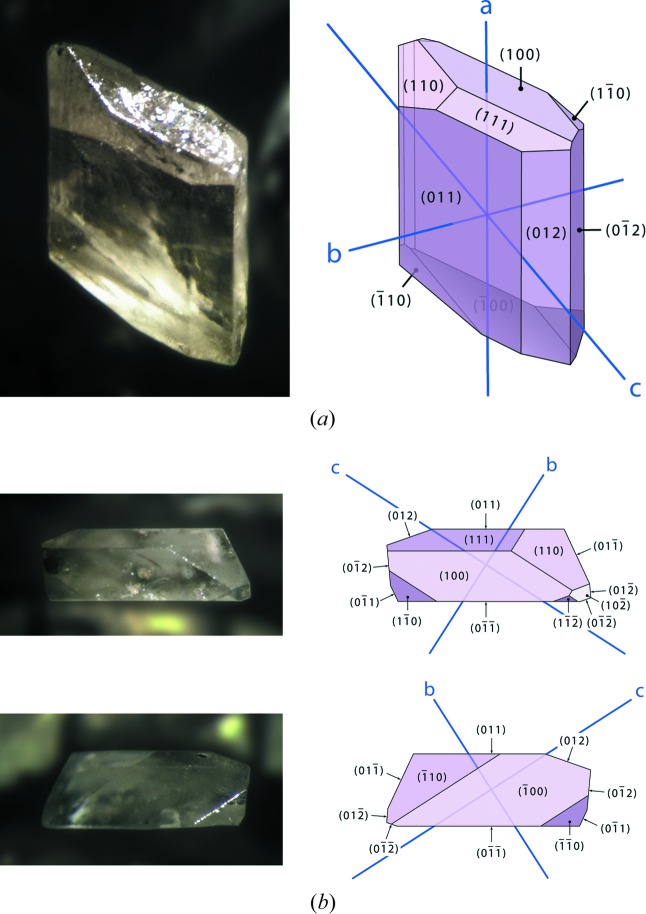
(*a*) Oblique microphotograph of a single crystal of MgSeO_4_·9H_2_O in air (left) and an indexed representation drawn using WinXmorph (right). (*b*) Images of the same crystal as shown in (*a*) viewed along the *a*-axis, and similarly indexed representations.

Magnesium selenate enneahydrate crystallizes in the monoclinic prismatic class as rhomboids that are tabular on (0 1 1) and exhibit marked growth sectors (Fig. 1 ▶). The facial indices shown in Fig. 2 ▶ were determined by X-ray diffraction methods and quantitative analysis of microphotographs, the morphology being modelled using *WinXmorph* (Kaminsky, 2005 ▶). The forms that dominate the crystals’ morphology are {0 1 1}, {0 1 2} and {1 1 0} prisms and the {1 0 0} pinacoid; much smaller {1 0 2} and {1 1 2} forms occasionally truncate the tips of the crystals.

**Figure 3:**
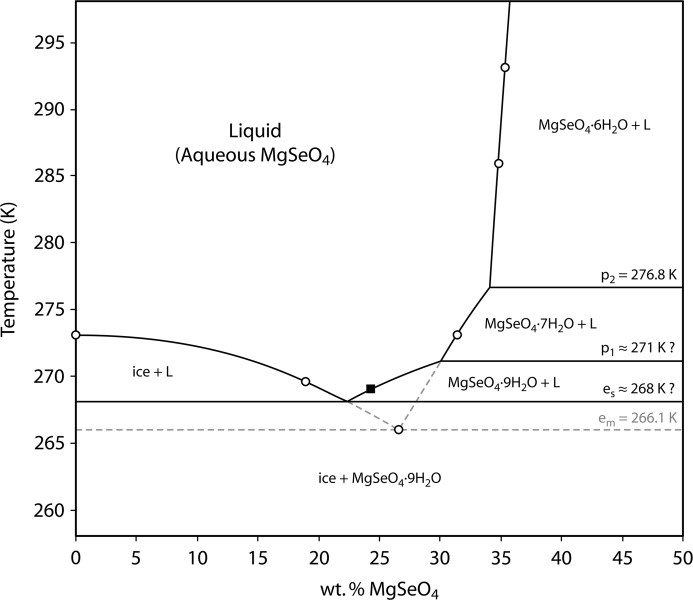
Reinterpretation of the binary MgSeO_4_–H_2_O phase diagram in light of the measured solubility of the enneahydrate at 269 K (filled square), observations of the incongruent melting behaviour of the undecahydrate and the apparent metastability of the heptahydrate at low temperatures. Open circles are measurements reported by Klein (1940 ▶) and solid lines are assumed to be the equilibrium liquidi and solidi; the dashed lines depict the previously understood low-*T* behaviour, which we now interpret as being metastable liquidi.

The salt content of the crystals was found by elementary thermogravimetry, after drying at ∼ 673 K for 24 h, to be 49.7 wt % MgSeO_4_ (mean of six independent determinations), which corresponds to 9.4 water molecules per formula unit. It is not unusual to find a small excess water content in these circumstances since crystals often occlude small pockets of mother liquor that are inevitably more water-rich. The dried residue was identified by X-ray powder diffraction as the β-phase of MgSeO_4_ (space group *Pbnm*), whilst elemental analysis using a Jeol JXA8100 microprobe confirmed the compound’s stoichiometry, the Mg:Se molar ratio being 1.03 (5). The solid content of the solution in equilibrium with these crystals was determined by drying at 673 K to be 24.3 wt % MgSeO_4_. This is significantly greater than the estimated solubility of MgSeO_4_·7H_2_O at the same temperature (*cf.* Klein, 1940 ▶), an observation that accords with other evidence we have obtained to suggest that the heptahydrate is actually metastable with respect to the enneahydrate over a narrow temperature range. Our determination of the enneahydrate’s solubility leads us to propose a modification of the binary phase diagram (Fig. 3 ▶) with a stable eutectic between MgSeO_4_·9H_2_O and ice around 2° above the previously reported eutectic between MgSeO_4_·7H_2_O and ice. The presumption that MgSeO_4_·9H_2_O undergoes incongruent melting to MgSeO_4_·7H_2_O + liquid remains to be experimentally verified.

### 2.2. Single-crystal neutron diffraction   

Two crystals were dried on filter paper and then loaded together into a thin-walled aluminium foil pouch suspended inside a 6 mm inner diameter vanadium sample tube. The sample holder was transported to the ISIS facility packed in dry ice and screwed onto the end of a cryostat centre-stick. This assembly was inserted into a closed-cycle refrigerator (CCR) on the SXD beamline (Keen *et al.,* 2006 ▶) after which the sample was cooled from ∼ 200 K directly to 5 K.

Time-of-flight Laue data were then collected in a series of four orientations, counting each for ∼ 2 h (300 µA of beam current). The diffraction spots from both crystals in the beam were indexed with the unit cell obtained at 250 K (Fortes, 2015 ▶), after which the intensities were extracted using the three-dimensional profile-fitting method implemented in *SXD*2001 (Gutmann, 2005 ▶). Data were subsequently collected using the same measurement strategy at 100, 175 and 250 K.

Structure refinement was carried out using *SHELX*2014 (Sheldrick, 2008 ▶; Gruene *et al.*, 2014 ▶) starting from the atomic coordinates – including estimated H positions – determined by the structure solution from X-ray powder diffraction data. The structure was found to be correct in all aspects and inspection of Fourier difference maps revealed no missing or incorrectly positioned atoms, and no evidence of possible H-atom site disorder, which the previous work had mooted as a possibility. For the 100, 175 and 250 K datasets, no restraints were used and all anisotropic displacement parameters were refined independently. At 5 K, the displacement ellipsoid of the Se atom became marginally non-positive definite (NPD), which is not wholly unsurprising given the very low temperature and the fact that this is the heaviest atom (by a factor of three) in the structure such that the displacement amplitudes are inevitably very small. This was remedied with the application of a *SHELX* instruction (ISOR) to force the selenium *U^ij^* to behave in a slightly more isotropic fashion.

### 2.3. Raman spectroscopy   

Laser-stimulated Raman spectra were measured using a portable B&WTek *i*-Raman Plus spectrometer equipped with a 532 nm laser (*P*_max_ = 37 mW at the probe tip) that records spectra over the range 171–4002 cm^−1^ with an optimal resolution of ∼ 3 cm^−1^. Measurements were carried out on a large single crystal of MgSeO_4_·9H_2_O in our cold room using the BC100 fibre-optic coupled Raman probe. Background noise was minimized by acquisition of multiple integrations, the time per integration being limited by detector saturation. At 262 K spectra were integrated for a total of 400 s (*P* = 18 mW). Another set of spectra were acquired on the same crystal after 5 months of storage in a freezer; this measurement at 259 K (1200 s, *P* = 37 mW) revealed no evidence of changes in hydration state. The temperature of the crystal was then reduced to 78 K by immersion in liquid nitrogen and another integration (1750 s, *P* = 37 mW) was acquired. Complementary measurements on single crystals of MgSeO_4_·6H_2_O were obtained at 262 K and on powders of MgSeO_4_ (β-phase formed at 673 K by dehydration of the enneahydrate) at room temperature.

### 2.4. Computational methods   

In order to confirm the veracity of our structure solution, and to aid in interpretation of the Raman spectrum, we carried out a first-principles calculation using density functional theory, DFT (Hohenberg & Kohn, 1964 ▶; Kohn & Sham, 1965 ▶), as implemented in the Vienna *ab initio* simulation package, *VASP* (Kresse & Furthmüller, 1996 ▶). The plane-wave expansion was treated using the projected augmented-wave method, PAW (Blöchl, 1994 ▶); with the PAW potentials generated by Kresse & Joubert (1999 ▶) and distributed with VASP. The exchange-correlation was accommodated using the PBE generalized gradient corrected functional (Perdew *et al.*, 1997 ▶). This form of the generalized gradient approximation (GGA) has been demonstrated to yield results of comparable accuracy to higher-level quantum chemical methods, such as MP2 and coupled-cluster methods, in hydrogen-bonded systems (*e.g.* Ireta *et al.*, 2004 ▶), despite not correctly representing dispersion forces.

Convergence tests were carried out to optimize the 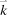-point sampling of the Brillouin zone within the Monkhorst–Pack scheme (Monkhorst & Pack 1976 ▶) and the kinetic energy cut-off of the plane-wave basis set. It was found that a 3 × 2 × 1 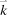-point grid combined with a kinetic energy cut-off of 875 eV yielded a total-energy convergence better than 10^−3^ eV per atom and pressure converged better than 0.1 GPa. A structural relaxation under zero-pressure athermal conditions was carried out, starting from the experimental crystal structure obtained at 10 K, in which the ions were allowed to move according to the calculated Hellman–Feynman forces and the unit-cell shape was allowed to vary. The relaxation was stopped when the forces on each atoms were less than 5 × 10^−4^ eV Å^−1^ and each component of the stress tensor was smaller than 0.05 GPa. The phonon spectrum was then computed using the small displacement method as implemented in the PHON code (Alfè, 2009 ▶). The construction of the full force-constant matrix requires knowledge of the force-field induced by displacing each atoms in the primitive cell in the three Cartesian directions. Since there are 132 atoms in the primitive cell, the total number of required displacements for this system would then be 792 (allowing for both positive and negative displacements), although this can be reduced to 198 by exploiting the symmetry elements present in the crystal. We used displacements of 0.02 Å, which are sufficiently small to obtain phonon frequencies that are converged to better than 0.1%. Since, in this instance, we are interested only in the normal modes at the Brillouin zone (BZ) centre, all of the required information could be obtained by computing the force matrix for the primitive unit cell.

The resulting modes at the BZ centre were classified according to the irreducible species of point group *C*_2*h*_; this was done using standard group-theory techniques with the help of the program *SAM* (Kroumova *et al.*, 2003 ▶), available at the Bilbao Crystallographic server (Aroyo *et al.*, 2006 ▶).

## 3. Results and discussion   

### 3.1. Description of the experimental structure and bonding   

**Figure 4:**
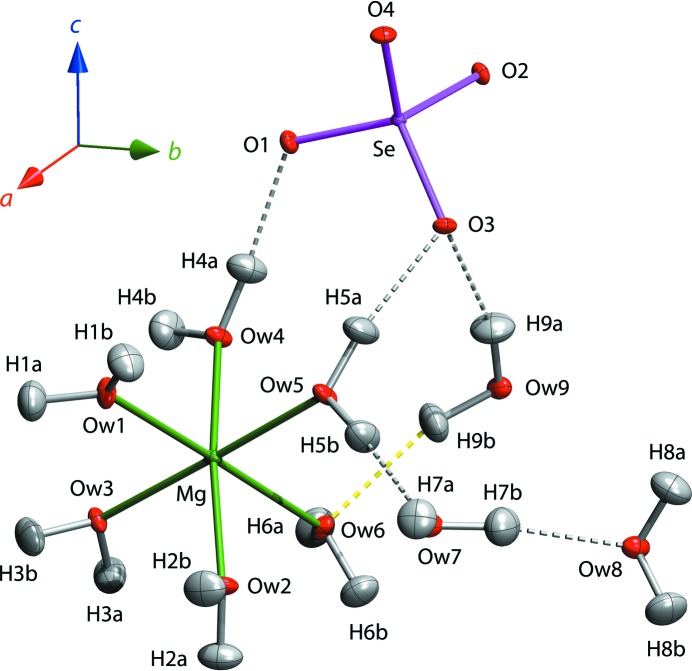
The asymmetric unit of MgSeO_4_·9H_2_O at 100 K with ellipsoids drawn at the 50% probability level. This and all subsequent structural representations are drawn using *DIAMOND* (Putz & Brandenburg, 2006 ▶).

The asymmetric unit of MgSeO_4_·9H_2_O is shown in Fig. 4 ▶; selected interatomic distances at various temperatures are listed in Tables 1–4 ▶ ▶ ▶ ▶. The more accurately determined atomic coordinates allow us now to make more definitive statements concerning the structure and its relationship to other crystalline hydrates.

The structure consists of isolated Mg(H_2_O)_6_^2+^ octahedra and SeO_4_^2−^ tetrahedra linked by a framework of moderately strong hydrogen bonds (with one exception discussed below, H⋯O ranges from 1.75 to 1.87 Å). The remaining three water molecules are not coordinated to either Mg or Se, occupying ‘voids’ between the polyhedral ions. The Mg(H_2_O)_6_^2+^ octahedron is slightly elongated along the Mg—O*w*6 vector compared with the other five Mg—O distances (Table 1 ▶), which is due to the tetrahedral coordination of O*w*6 compared with the trigonal coordination of the remaining Mg-coordinated water molecules. Conversely, the Se—O1 bond is significantly shorter than the other three Se—O distances, which is due to O1 acting as an acceptor of two hydrogen bonds whilst O2 through to O4 accept three hydrogen bonds each (Tables 1 ▶ and 3 ▶). The same pattern of systematic bond-length variations occurs in both MgSeO_4_·7H_2_O (Fortes & Gutmann, 2014 ▶) and MgSeO_4_·6H_2_O (Kolitsch, 2002 ▶), as well as their sulfate analogues (Baur, 1964 ▶; Ferraris *et al.*, 1973 ▶; Fortes *et al.*, 2006 ▶), depending on the number of hydrogen bonds accepted by a given water molecule or O atom (Table 2 ▶). Indeed, such patterns provide useful complementary information of value in deducing the geometry of an otherwise indeterminate hydrogen-bond network from, for example, powder X-ray diffraction data.

**Figure 5:**
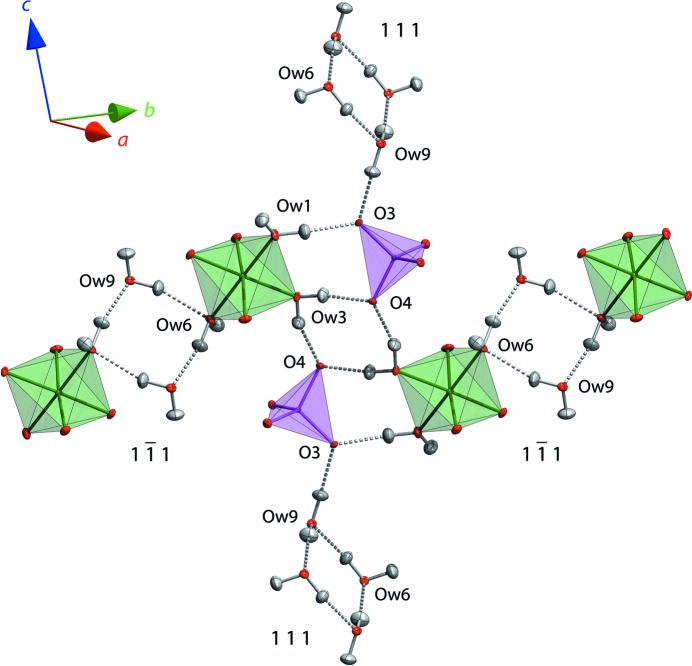
The two symmetry-inequivalent square rings that occur in the structure of MgSeO_4_·9H_2_O and the manner in which these bridge to form chains of octahedra (horizontally, along [1 1 0]) and chains of tetrahedra (vertically, along [1 0 1]).

An interesting aspect of the supramolecular connectivity is the occurrence of two symmetry-inequivalent four-sided rings (Fig. 5 ▶). One of these is a water tetramer defined by O*w*6 and O*w*9, which lies in the fourfold family of {1 1 1} planes, located on centres of symmetry at ½, ½, 0 and ½, 0, ½; the second square ring is defined by O*w*3 and the sulfate oxygen O4, which lie very close to the pair of planes (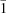
 2 2) and (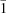 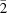
 2), again on inversion centres at 0, 0, 0 and 0, ½, ½. The second of these two sets of rings, by virtue of donating hydrogen bonds to sulfate O atoms, is obliged to be ordered. However, the first ring is not so obliged and it was hypothesized by Fortes (2015 ▶) that occupational disorder of the H atoms could occur in ring #1 in a manner similar to that which occurs in the water tetramer in Na_2_SO_4_·10H_2_O (*cf.* Brand *et al.*, 2009 ▶, and references therein). In sodium sulfate decahydrate, variable ordering of the hydrogen bonds in the square rings has a measureable influence on the material’s thermal expansion depending on the cooling rate: high cooling rates freeze in substantial disorder, resulting in a strained unit cell at low temperatures.

One feature that led to the inference of possible disorder in ring #1 was the presence of a rather long (*i.e.* weak) hydrogen bond between O*w*9 and O*w*6, roughly 4–8% longer than the other O⋯O contacts, which was apparent in the original X-ray powder refinement (Fortes, 2015 ▶). This long hydrogen bond is confirmed by the single-crystal data, the O*w*9⋯O*w*6 distance through H9*B* increasing from 2.923 (4) Å at 5 K to 2.984 (14) Å at 250 K; compare these with the value of 3.05 (1) Å obtained from X-ray powder data at 248 K. Additionally, the ADPs of H atoms associated with the three interstitial water molecules (O*w*7, O*w*8 and O*w*9) are, in general, of greater magnitude than of the Mg-coordinated waters and the ADP of H9*B* is either close to, or is, the largest of all H-atom ADPs at each of the four temperatures. H9*B*⋯O*w*6, indicated by a dashed yellow rod in Fig. 4 ▶, thus *appears* to be the weakest hydrogen bond in the structure.

**Figure 6:**
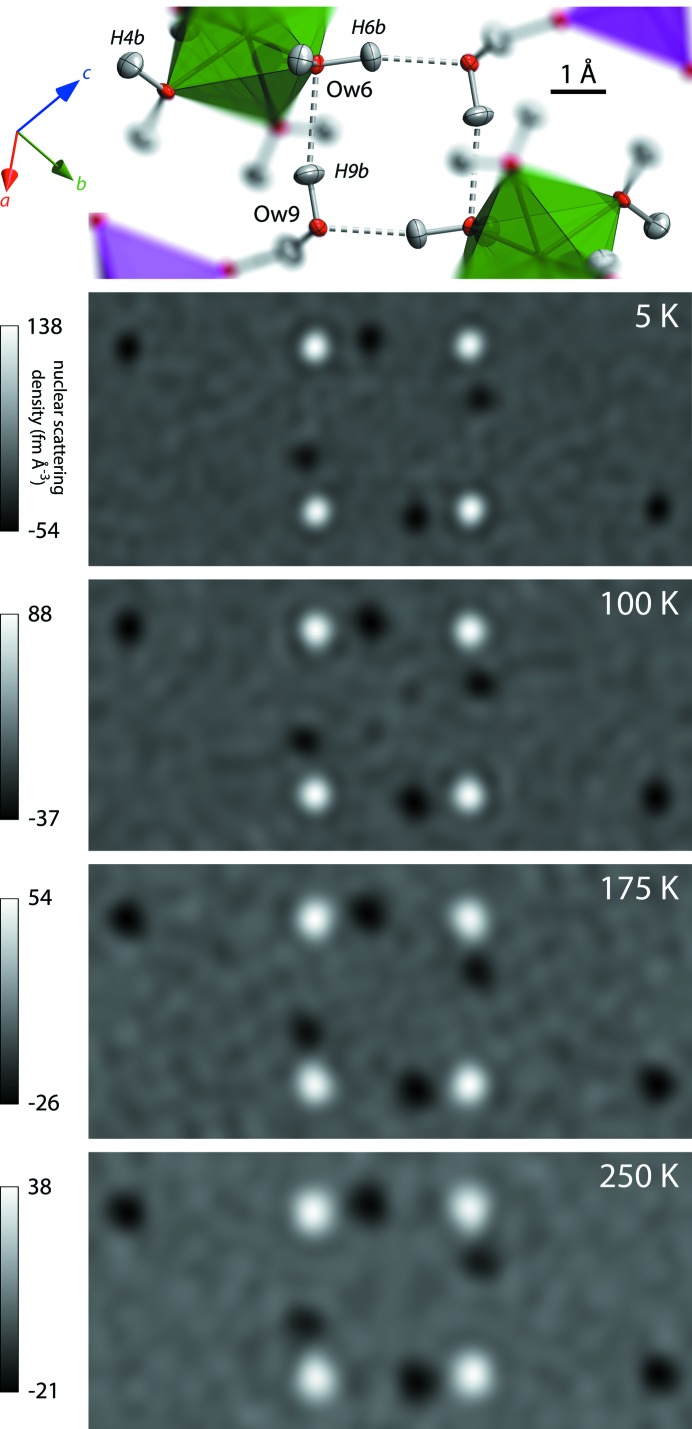
Fourier maps, *F*_obs_, phased on the refined structure at each temperature and visualized in a slice through the water tetramer defined by O*w*6 and O*w*9 (the panel at the top depicts the corresponding local atomic structure). Peaks – bright spots – locate O atoms and holes – dark spots – the H atoms. Evidently, the H-atom sites in the plane of the ring are fully occupied at all temperatures. Fourier maps are generated in *VESTA* (Momma & Izumi, 2011 ▶).

As shown in Fig. 6 ▶, Fourier maps (*F*_obs_) phased on the refined structure reveal no partially occupied H-atom sites in the tetramer rings at any temperatures, at least down to the level of the background noise (around 5% of the nuclear scattering density due to a H atom); the O*w*6 and O*w*9 molecules are thus orientationally ordered and remain so throughout cooling and warming.

**Figure 7:**
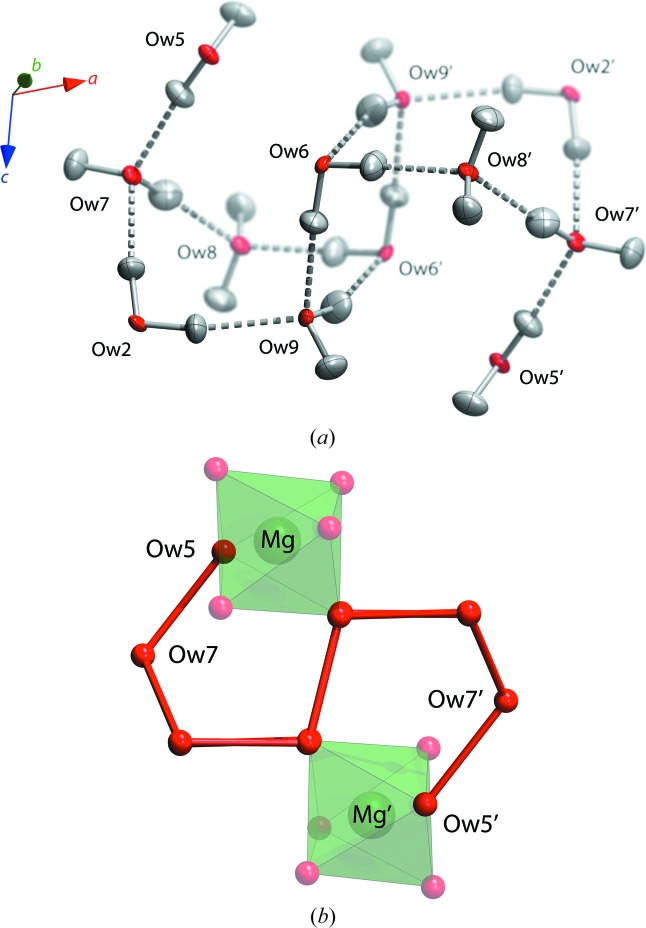
(*a*) The hydrogen-bonded dodecamer of water molecules viewed obliquely and (*b*) perpendicular to the central tetramer unit illustrating the pentagonal sigmoidal folding of the structure and its relationship to the adjacent Mg(H_2_O)_6_ octahedra.

One hitherto unidentified aspect of the structure of these highly hydrated inorganic salts is the occurrence of extended water polymer networks. In MgSeO_4_·9H_2_O the water tetramer described above is merely the centre of a larger structure, a centrosymmetric dodecamer, (H_2_O)_12_, comprised of O*w*2, O*w*5, O*w*6, O*w*7, O*w*8 and O*w*9 (Fig. 7 ▶ *a*). The aforementioned tetramer is extended by a pair of pentagonal rings with apical chains folded back upon the structure in such a way as to form a pseudo-pentagonal sigmoidal profile (Fig. 7 ▶ *b*), the ‘missing’ edge of the open pentagon being supplied by the edge of the Mg(H_2_O)_6_ octahedron.

**Figure 8:**
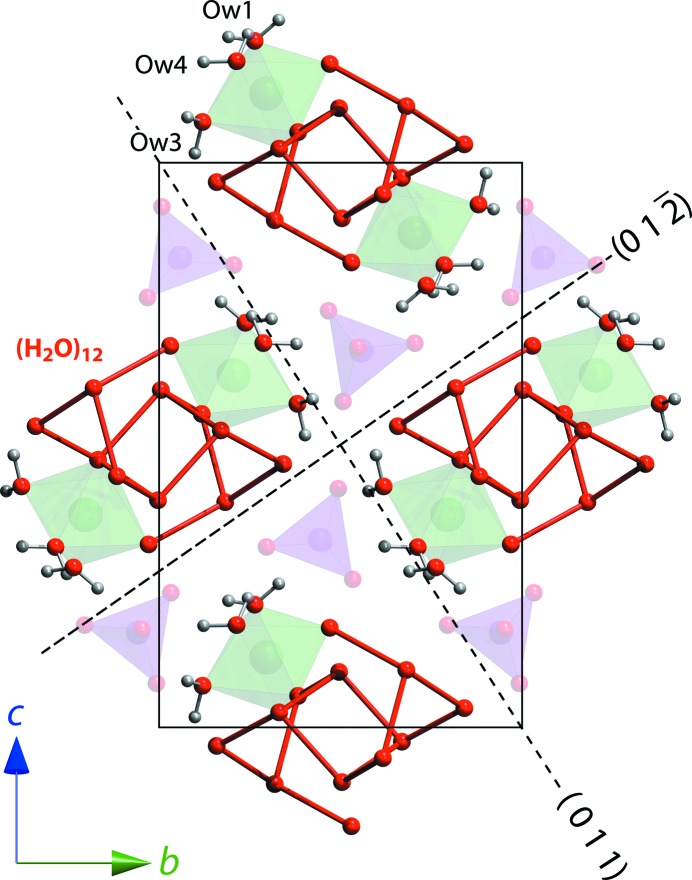
The broader context of the neutral dodecamer’s relationship to the ionic polyhedra and to particular planes that were identified previously as common forms in the macroscopic morphology of the crystals.

The position of the neutral dodecamer cluster in relation to its surrounding ionic polyhedra and the remaining three water monomers is shown in Fig. 8 ▶.

**Figure 9:**
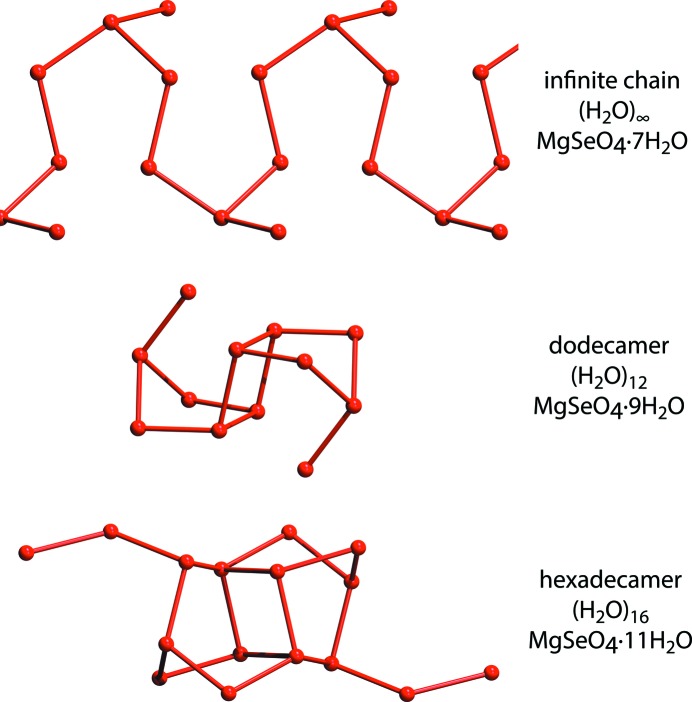
Polymeric water structures found in this and related hydrate structures revealing the propensity of these chains or clusters to adopt pentagonal motifs.

Similar pentagonal motifs occur in the polymeric water frameworks of all related hydrates with one or more free interstitial water molecule (Fig. 9 ▶). In MgSeO_4_·7H_2_O there is an infinite chain of open-sided pentagons with decorated corners extending along the crystal’s *c*-axis, whereas in MgSeO_4_·11H_2_O there is a hexadecamer, (H_2_O)_16_, composed of closed pentagonal rings, which has short side-chains extending along the *c*-axis of the crystal.

The observation of dodecamer and hexadecamer water clusters in crystal structures is not unique (see Song & Ma, 2007 ▶; Wang *et al.*, 2010 ▶; Li *et al.*, 2012 ▶; Ghosh & Bharadwaj, 2004 ▶; Jin *et al.*, 2008 ▶); indeed there is a dodecamer structure very similar to that described here in copper citrate phenanthroline hexahydrate (Fu *et al.*, 2010 ▶). Close examination of their structure, however, reveals that the two pentagons of the dodecamer appear not to be hydrogen bonded to one another. Consequently, their ‘novel’ self-assembled structure may not be a true 12-mer, whereas ours is a bona fide hydrogen-bonded polymeric unit.

The dominance of pentagonal motifs in these water polymer structures is particularly interesting since five-sided rings have been implicated in the unusual properties of supercooled water (Speedy, 1984 ▶) and they are a key structural feature of various high-pressure forms of ice, such as ices III, IX, XII and XIV (Londono *et al.*, 1993 ▶; Lobban *et al.*, 1998 ▶; Salzmann *et al.*, 2006 ▶), as well as the clathrate hydrates (*e.g.* Kirchner *et al.*, 2004 ▶). Furthermore, pentagonal motifs are found in hydrogen-bonded ‘ices’ such as ammonia hydrates (Fortes *et al.*, 2009 ▶); hence the importance of solutes in aqueous solutions (or the substrate; Carrasco *et al.*, 2009 ▶) as a structure-forming template cannot be overlooked.

### 3.2. Computational analysis of the structure and bonding   

**Figure 10:**
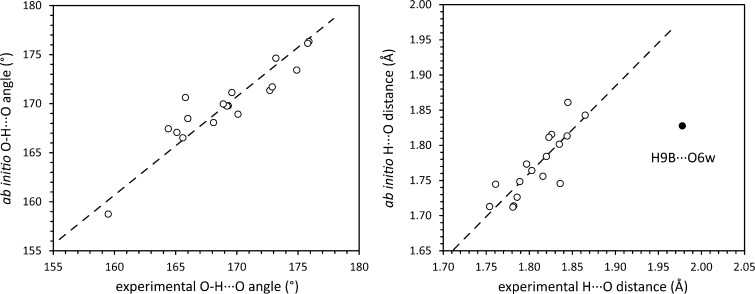
Correlation between the experimentally determined and calculated O—H⋯O bond angles (left) and H⋯O bond lengths (right). The filled circle on the right represents the H9*B*⋯O*w*6 bond, which appears to be significantly weaker and longer than other water–water hydrogen bonds in the structure.

The *ab initio* zero-pressure athermal relaxed structure agrees very well, for the most part, with the experimental structure described above. The unit-cell dimensions are *a* = 7.28013, *b* = 10.50519, *c* = 17.25609 Å, β = 109.2550° and *V* = 1245.907 Å^3^, values that are each within 1% of the experimental unit cell at 5 K. Fractional atomic coordinates are given in §S2 of the supporting information. Several noteworthy differences between calculations and experiments appear in Tables 1 ▶ and 3 ▶. Firstly, the calculated Se—O and O—H bond lengths are overestimated by ∼ 2%; nonetheless, the pattern of shorter and longer Se—O distances corresponds with the number of hydrogen bonds donated to these atoms in the same sense as noted previously. Secondly, the Mg—O bond lengths are similarly overestimated, albeit by ∼ 1%; however, there is a strong correlation between the calculated and experimental Mg—O distances (*R* ^2^ = 0.990). By contrast, the calculated H⋯O hydrogen bond lengths are almost all shorter than those observed in the real crystal. Evidently, the over-inflation of the unit cell by the greater Se—O and Mg—O distances is largely cancelled out by the shorter hydrogen bonds. There is, again, a good linear correlation (*R* ^2^ = 0.827) between hydrogen-bond angles and, with a single exception, a reasonable correlation (*R* ^2^ = 0.703) between H⋯O distances from DFT calculations and from the real crystal (Fig. 10 ▶). The outlier in this instance is one of the bonds comprising the water tetramer, H9*B*⋯O*w*6: DFT calculations give a length of 1.8275 Å for this contact, very similar to other hydrogen-bonded distances in the structure; however, this differs markedly from the length found in our single-crystal neutron diffraction refinements (∼ 1.98 Å) and inferred from the X-ray powder diffraction refinements (∼ 2.05 Å).

It becomes a matter of interest, therefore, to ascertain the energetic properties of the hydrogen bonds in the structure. The properties of the hydrogen bonds may be described quantitatively from the topology of the electron density according to Bader’s Quantum Theory of Atoms-in-Molecules, QTAIM (Bader, 1990 ▶). Of interest to us are the saddle points where the gradient in the electron density, ∇ρ(*r*), vanishes, the so-called bond critical points (BCPs). Useful metrics of the bond strength and character are the electron density at the BCP, ρ(*r*_BCP_), and the Laplacian of the electron density at the BCP, ∇^2^ρ(*r*_BCP_), which represents the 3 × 3 Hessian matrix of second partial derivatives of the electron density with respect to the coordinates. The eigenvalues of this matrix, λ_1_, λ_2_ and λ_3_ (which sum to ∇^2^ρ) are the principal axes of ‘curvature’ of the electron density perpendicular to the bond (λ_1_, λ_2_) and along the bond (λ_3_). A negative Laplacian at the bond critical point generally corresponds to a concentration of electron density, which is characteristic of a covalent bond, whereas ionic bonds and hydrogen bonds have a positive Laplacian, indicative of a depletion in electron density. We have used the program *AIM-UC* (Vega & Almeida, 2014 ▶) to compute the locations and properties of the electron density at the H⋯O BCPs in magnesium selenate enneahydrate (Table 5 ▶).

The dissociation energy of the hydrogen bond may be estimated with varying degrees of accuracy from the electron density (*e.g.* Vener *et al.*, 2012 ▶). The total energy density is the sum of the local kinetic and potential electronic energies, *G*(*r*) and *V*(*r*), respectively, at the BCP (Bader & Beddall, 1972 ▶)
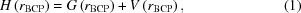
where the potential energy is related to the Laplacian of the electron density *via* the local form of the virial theorem (Bader, 1990 ▶)
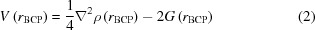
and the kinetic energy is obtained by partitioning of the electron density (*e.g.* Abramov, 1997 ▶)
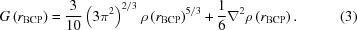
Espinosa *et al.* (1998 ▶) proposed that the hydrogen-bond energy, *E*_HB_, could be obtained simply from the potential energy density
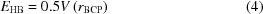
and this expression continues to be used widely, whereas Mata *et al.* (2011 ▶) subsequently suggested that a more accurate value could be found from the kinetic energy density
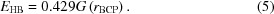
A subsequent analysis by Vener *et al.* (2012 ▶) found that equation (4) systematically overestimates *E*_HB_ compared with the spectroscopically determined hydrogen-bond energies. However, the value given by equation (5) appears to yield reasonably accurate values of *E*_HB_. In Table 6 ▶ we detail *E*_HB_ as calculated using equations (4) and (5). Furthermore, we give a ‘corrected’ value of *E*_HB_ based on equation (4) and the tabulated results in Vener *et al.* (2012 ▶) such that *E*_HB_(corrected) = 0.465*E*_HB_ + 16.58. The mean values of *E*_HB_ in the right-hand column thus represent our most accurate determination of the hydrogen-bond dissociation energy in this compound.

As shown in Table 4 ▶, the hydrogen bonds in these hydrates may be classified according to the donor and acceptor species, which of itself reveals interesting trends in the ways these structures are organized with increasing hydration state. Notably, the interstitial water molecules that occur once the cation is ‘saturated’ serve more often than not to bridge between the cationic and anionic polyhedra instead of forming hydrogen bonds with other interstitial water molecules. Categorization of the hydrogen-bond energies in Table 6 ▶ according to the types listed in Table 4 ▶ shows that the weakest hydrogen bonds (mean = 32.4 ± 3.3 kJ mol^−1^) are those between Mg-coordinated waters and sulfate O atoms. The hydrogen bonds donated by free interstitial waters to the sulfate O atoms are similarly weak, 32.9 ± 2.5 kJ mol^−1^. On the other hand, hydrogen bonds formed between water molecules are significantly stronger; those between Mg-coordinated water and interstitial water have a mean energy of 39.0 ± 2.3 kJ mol^−1^ and the single hydrogen bond between two interstitial waters has an energy of 36.7 kJ mol^−1^. The exception, again, is the H9*B*⋯O*w*6 bond; whilst this is not quite the weakest hydrogen bond in the structure it is much the weakest (by ∼ 25%) of the water–water hydrogen bonds.

### 3.3. The Raman spectrum   

MgSeO_4_·9H_2_O crystallizes in the centrosymmetric space group *P*2_1_/*c* having a primitive cell with *C*_2*h*_ point-group symmetry and four formula units per unit cell; all ions and molecules are located on sites of *C*_1_ symmetry. Based on a consideration of the normal vibrational modes of the ionic polyhedra and the neutral water molecules, we have carried out a factor group analysis by the correlation method to determine the symmetry species of all Raman-active modes. Allowing for the modes corresponding to translation of the entire crystal, we find that there are 198 normal modes summarized as Γ_opt_(Raman) = 99 *A_g_* + 99*B*_1*g*_.

**Figure 11:**
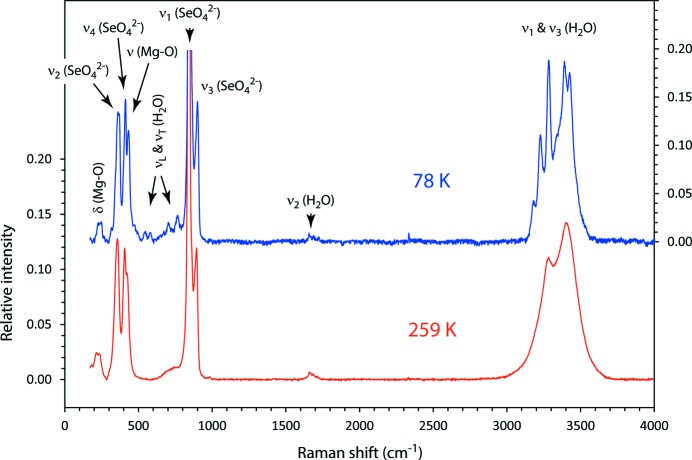
Raman spectrum of MgSeO_4_·9H_2_O at 259 and 78 K, measured on the (0 1 1) face of a single crystal. Pertinent groups of vibrational modes contributing to the observed features are labelled. The raw data are given in Table S3 of the supporting information.

**Figure 12:**
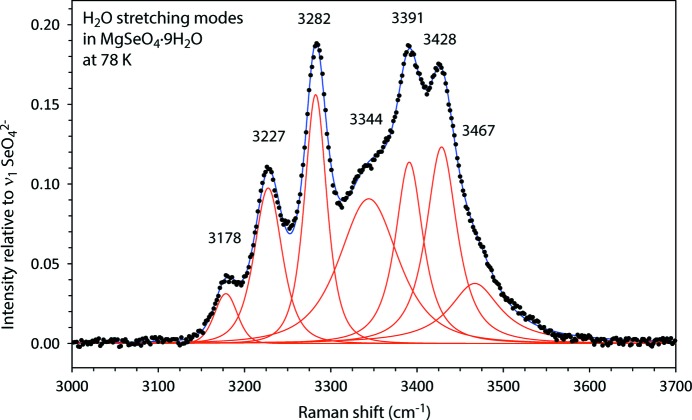
Expended view of the high frequency region of the Raman spectrum of MgSeO_4_·9H_2_O with fitted Lorentzian components depicted in red.

The majority of these vibrations are associated with the water molecules, grouped into three distinct portions of the spectrum (Fig. 11 ▶). The highest frequency modes, observed between ∼ 3100–3600 cm^−1^, correspond with the O—H symmetric and asymmetric stretch, ν_1_ and ν_3_, respectively. At higher temperatures, the asymmetric potential well in which the H atoms are oscillating leads to thermal broadening of the peaks, although it is nonetheless possible to identify five discrete Lorentzian contributions to the high-frequency feature. When the temperature is reduced to 78 K, however, the peaks sharpen substantially, making it possible to clearly identify at least seven individual bands (Fig. 12 ▶).

In the mid-frequency range, around 1600–1700 cm^−1^, a rather weak feature, comprising three identifiable peaks, is produced by the symmetric bending mode, ν_2_, of water. In the low-frequency range, below 1000 cm^−1^, there are numerous librational and rotational modes of water but these are so weak and diffuse that they are manifested (at 259 K) as rather broad ‘bumps’ between the much sharper and more intense selenate peaks. As with the stretching modes, cooling results in sharpening of these features such that discernible peaks appear between ∼ 540 and 770 cm^−1^ (see Fig. 11 ▶, upper spectrum, and Table 7 ▶).

Below 1000 cm^−1^, the dominant source of Raman scattering is the tetrahedral SeO_4_^2−^ ions. The ideal SeO_4_^2−^ exhibits *T_d_* point-group symmetry with four internal vibrational modes: the symmetric Se—O stretch, ν_1_(*A*_1_); the doubly degenerate symmetric deformation modes, ν_2_(*E*); the triply degenerate asymmetric stretch, ν_3_(*F*_2_); and the triply degenerate asymmetric deformation modes, ν_4_(*F*_2_). The site symmetry of the SeO_4_^2−^ ions in the crystal (*C*_1_) break the degeneracy of these internal modes, leading to nine modes of symmetry *A*. Under the factor-group symmetry of the unit-cell (*C*_2*h*_), each of these nine components are further split into modes of symmetry *A* *_g_*, *B* *_g_*, *A_u_* and *B_u_*, of which only the first two are Raman active. Consequently, we would expect to find 9*A_g_* and 9*B_g_* vibrational modes in the Raman spectrum due to the SeO_4_^2−^ ion. The extent to which nearly degenerate bands are dispersed depends on the distortion of the selenate ion from ideal *T_d_* symmetry.

The highly symmetric ν_1_(*A_g_*) mode of SeO_4_^2−^ is the sharpest and most intense feature, occurring at 841.3 cm^−1^ at 259 K and shifting to 845.9 cm^−1^ at 78 K (compare with 844.1 cm^−1^ in MgSeO_4_·6H_2_O). The asymmetric stretching modes are at a slightly higher frequency, the six expected components contributing to the scattering between ∼ 850 and 900 cm^−1^, which can be deconvolved into three broad bands (Table 7 ▶). The spread of band centres for these ν_3_ modes, Δν_3_, are ∼ 35 cm^−1^ in the enneahydrate and 43 cm^−1^ in the hexahydrate; these differences are attributable to the difference in Se—O bond lengths noted in Table 2 ▶, which are due to the number of hydrogen bonds donated to each apical oxygen. These shifts match the general trend observed in both Mg- and Fe-sulfate hydrates of a shift in ν_1_ to lower frequency and a smaller dispersion of ν_3_ with increasing hydration number (Wang *et al.*, 2006 ▶; Chio *et al.*, 2007 ▶). Similar trends have been found in *ab initio* calculations of hydrated tetrahedral oxyanions (Pye & Walker, 2011 ▶).

Predictably, given the overestimate of the *ab initio* Se—O bond length, the calculated vibrational frequencies differ substantially from the observations; ν_1_(*A_g_*) = 783.34 cm^−1^ and ν_1_(*B_g_*) = 783.01 cm^−1^. Similarly, the calculated asymmetric stretching frequencies fall between 789.14 and 843.15 cm^−1^, red-shifted by 50–70 wavenumbers.

The four components of ν_2_ are coalesced into a single broad band centred at 352.6 cm^−1^, whilst ν_4_ appears as a band at 403.1 cm^−1^. Once again, cooling blue-shifts the band centres by around 10 cm^−1^. The observed vibrational frequencies of the selenate ion in MgSeO_4_·9H_2_O are very similar to those of the fully hydrated free ion in aqueous solution, for which ν_1_ = 837 cm^−1^, ν_2_ = 348 cm^−1^, ν_3_ = 875 cm^−1^ and ν_4_ = 414 cm^−1^ (Walrafen, 1963 ▶), but differ systematically from those we have obtained for MgSeO_4_·6H_2_O and β-MgSeO_4_, and from other anhydrous and hydrated selenate crystals reported previously (Scheuermann & Schutte, 1973 ▶; Berger, 1976 ▶; Ti *et al.*, 1976 ▶; Park & Frech, 1989 ▶; Wildner *et al.*, 2004 ▶). In other words, the selenate ion in the 9-hydrate is closer than the hexahydrate to ideal *T_d_* symmetry, which supports the crystallographic analysis presented earlier.

The remaining low-frequency features, specifically a peak at 425 cm^−1^ and a pair of overlapping peaks at 210–235 cm^−1^, are assigned to stretching and deformation of the Mg(H_2_O)_6_ octahedra. The Raman spectroscopic data are given in §S3 of the supporting information.

### 3.4. Crystal morphology   

Examination of Fig. 8 ▶ reveals a straightforward rationale for the dominance of {0 1 1} and {0 1 2} forms since these are planes across which hydrogen bonds are donated to selenate O atoms; recall from §3.2 that these are weaker than the water–water hydrogen bonds. The {0 1 1} planes correspond with the triangular face of the Mg(H_2_O)_6_ octahedra defined by the three monomeric waters, O*w*1, O*w*3 and O*w*4 and the {0 1 2} planes have the highest density of octahedra.

**Figure 13:**
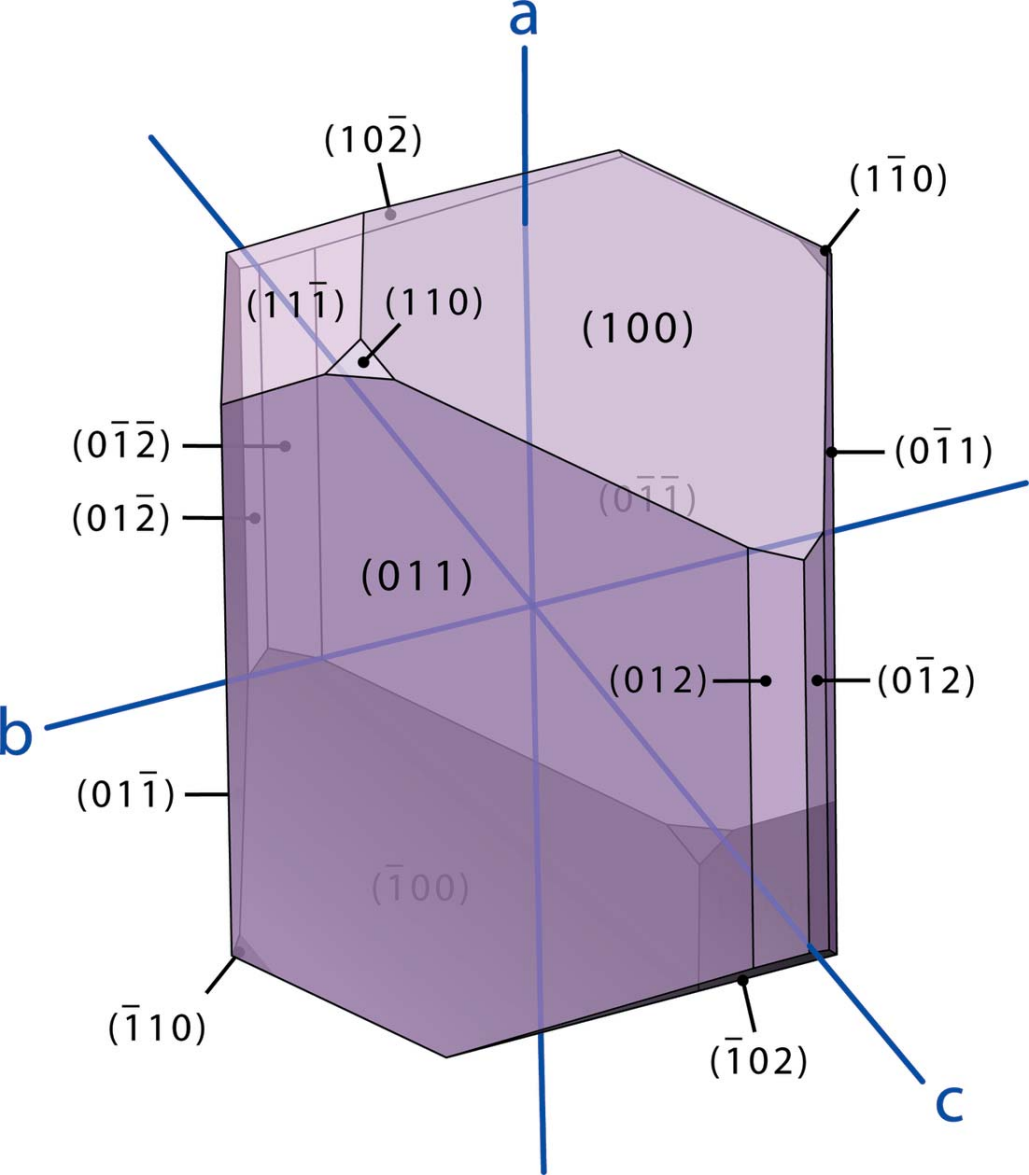
Morphology of the MgSeO_4_·9H_2_O crystal calculated using the BFDH method in *WinXMorph* and depicted in the same orientation as the crystal photographed in Fig. 1 ▶.

As shown in Fig. 13 ▶, a fair approximation of the crystal’s morphology can be obtained by a more straightforward analysis based on interplanar spacing density, namely the Bravais–Friedel–Donnay–Harker, BFDH, model (Bravais, 1866 ▶; Friedel, 1907 ▶; Donnay & Harker, 1937 ▶): we have used the implementation of this model coded in *WinXMorph* (Kaminsky, 2007 ▶). Note that the predicted crystal has a blockier habit; however, our crystals were grown on the bottom of petri dishes and it seems likely that they would be less platy if they were to be grown suspended in solution. Nevertheless, the predicted forms agree well with those observed in Figs. 1 ▶ and 2 ▶.

### 3.5. Behaviour on warming   

Determination of the unit-cell parameters at four temperatures between 5 and 250 K (Table 8 ▶) allows some initial comments to be made with respect to the magnitude and anisotropy of the thermal expansion. All three crystallographic axes expand normally on warming, albeit to substantially different degrees; the *b*-axis expands very little and the *c*-axis expands rather more (0.019 *versus* 0.062 Å, respectively, in absolute terms). The monoclinic angle β is apparently saturated below ∼ 100 K and then shrinks marginally (by 0.15°) on warming to 250 K.

Since we judge there to be comparatively little ambiguity in the thermal expansion at 150 K, which is not necessarily the case at low temperatures (where β saturates) or at high temperatures (where the behaviour of the *b*-axis is unclear), we may be reasonably confident in the accuracy (if not the precision) of the derived magnitudes and orientation of the thermal expansion tensor’s principal axes. At 150 K, a simple point-by-point derivative yields α_1_ ≃ 34 ± 10 × 10^−6^ K^−1^, α_2_ ≃ 12 ± 11 × 10^−6^ K^−1^, α_3_ ≃ 16 ± 10 × 10^−6^ K^−1^, and the volume thermal expansion, α_V_ ≃ 58 ± 18 × 10^−6^ K^−1^. Whilst α_2_ is obliged by convention to be parallel with the *b*-axis, α_1_ and α_3_ are free to adopt any orientation they wish in the *ac* plane, whilst remaining orthogonal to one another; we find that α_3_ is tilted by just over 20° from the *c*-axis. Although the precision of these expansion coefficients is poor, it is clear that the structure expands highly anisotropically and that the magnitude of α_V_ is similar to that of other water-rich salt hydrates.

Short X-ray powder diffraction scans were collected from MgSeO_4_·9H_2_O at nine temperatures on warming from 251.4 to 298.5 K (10–35° 2θ, 15 min each) in order to evaluate the thermal stability of the material through its expected dissociation point and to identify any products of its (partial) melting. This was done by crushing single crystals of MgSeO_4_·9H_2_O with an LN_2_-cooled steel pestle and mortar and loading the powder into a Peltier-cooled X-ray cold stage (Wood *et al.*, 2012 ▶) at ∼ 260 K. The operation of this stage allows fairly accurate control of the sample temperature in the range from 250 to 300 K (depending on the ambient temperature inside the diffractometer enclosure) by the simple expedient of adjusting the power supplied to the Peltier element. Unexpectedly, MgSeO_4_·9H_2_O persisted up to 298.5 K, albeit with significant variations in some peak shapes – including some slight splitting – and intensities that are attributable to changes in the surface texture of the specimen. Overall, the sample spent 30 min in air at temperatures ≥ 290 K with no obvious signs of transforming to another hydrate. Further work will be necessary to determine the material’s behaviour in aqueous solution above 270 K and in air above 300 K.

## 4. Comparisons to related compounds   

Amongst the *M* ^2+^ *X*O_4_·*n*H_2_O compounds, this is the first enneahydrate to have its structure determined; enneahydrates in such materials were wholly unknown until very recently. The occurrence of MgSeO_4_·9H_2_O in the MgSeO_4_–H_2_O system is perhaps less surprising than in the analogous MgSO_4_–H_2_O system since a *hemi*-enneahydrate, [Mg(H_2_O)_4_(SeO_4_)]_2_·H_2_O, was identified by Klein (1940 ▶) and its structure determined by Krivovichev (2007 ▶). What is more surprising, given the extensive degree of isotypism amongst *M* ^2+^ *X*O_4_·*n*H_2_O compounds, is that magnesium selenate enneahydrate has a different unit-cell metric (and presumably a different structure) to magnesium sulfate enneahydrate, although both are monoclinic crystals. Nonetheless, there are precedents; the *M* ^2+^(NO_3_)_2_·6H_2_O series of compounds exhibit a remarkable diversity, not only of symmetry but also supramolecular connectivity, even between substances where the cation radii are quite similar (Ferrari *et al.*, 1967 ▶; Braibanti *et al.*, 1969 ▶; Bigoli *et al.*, 1971 ▶).

Amongst inorganic substances, true enneahydrates – by which we mean materials in which the cation to water ratio is one to nine – are comparatively rare; known examples fall into several distinct groups with various types of cation coordination polyhedra and different degrees of water polymerization.

### 4.1. Ninefold coordinated trivalent cations (*r*_ionic_ > 1 Å)   

Compounds with the general formula *M* ^3+^(*X* ^−^)_3_·9H_2_O, where *M* is a lanthanide element and *X* is a halogen element (James *et al.*, 1927 ▶; Sokolova *et al.*, 1986 ▶; Timofte *et al.*, 2005*a* ▶,*b* ▶) or halogen oxyanion, typically BrO_3_^−^ (Poulet *et al.*, 1975 ▶; Gallucci *et al.*, 1982 ▶; Gerkin & Reppart, 1987 ▶; Abbasi & Eriksson, 2006 ▶). The cation is ninefold coordinated by water to form a tricapped trigonal prism: the water molecules are isolated monomers, forming hydrogen bonds solely with the anions. Since the cation radius varies only slightly from approximately 1.05 to 1.20 Å across the lanthanide series, all known examples of this class of hydrates are isotypic.

### 4.2. Ninefold coordinated tetravalent cations   

Compounds with the general formula *M* ^4+^(*X*O_4_^2−^)_2_·9H_2_O, which have examples where *M* may be Th or U, and *X* may be S or Se. These materials have, for the most part, not been well characterized crystallographically; the older literature reports goniometric measurements (Topsøe, 1874 ▶; Fock, 1900 ▶; Krause, 1901 ▶), but the non-H structure of Th(SO_4_)_2_·9H_2_O was only determined quite recently (Albrecht *et al.*, 2011 ▶). In this instance the cation is ninefold coordinated by seven water molecules and two sulfate O atoms, leaving two interstitial lattice water molecules.

### 4.3. Sixfold coordinated trivalent cations (*r*_ionic_ < 0.65 Å)   

Compounds of the general formula *M* ^3+^(*X* ^−^)_3_·9H_2_O, where examples are known with *M* = Fe, Al, Cr and Ga, and *X* = Br^−^, NO_3_^−^ or ClO_4_^−^ (Hair & Beattie, 1977 ▶; Hermansson, 1983 ▶; Gubrynowicz & Strömich, 1987 ▶; Lazar, Ribár, Divjaković & Mészáros, 1991 ▶; Lazar, Ribár & Prelesnik, 1991 ▶; Hendsbee *et al.*, 2009 ▶; Schmidt *et al.*, 2014 ▶; Hennings *et al.*, 2014*a* ▶,*b* ▶). The cation in these materials is sixfold coordinated by water, forming isolated octahedra, leaving three interstitial water molecules. The presence of several interstitial water molecules provides opportunities for the formation of polymerized structures, the size and complexity of which are dictated largely by the geometry of the anion. The perchlorates form simple trimers, (H_2_O)_3_, whereas the nitrates form an octamer (H_2_O)_8_ comprised of a decorated square ring, as well as having trimers and monomers; the bromides form the largest polymeric units in this class, consisting of branched (H_2_O)_9_ chains.

### 4.4. Poly-coordinated divalent cations (*r*_ionic_ 0.7–1.3 Å)   

These have the general formula *M* ^2+^(*X* ^−^)_2_·9H_2_O. Crystal structures have been determined for examples where *X* may be Br^−^, I^−^ and ClO_4_^−^ (Hennings *et al.*, 2013 ▶, 2014*c* ▶; Schmidt *et al.*, 2014 ▶). There is, however, a group of divalent metal *nitrate* enneahydrates with as-yet unknown structures, which includes Mg, Fe, Co, Cu, Ni, Zn and Cd (*e.g.* Funk, 1899 ▶); the fact that these substances typically crystallize from solution at or below 260 K is a fair explanation for the lack of any impetus to study their structures. Similarly, perchlorate enneahydrates of both Ni and Co have been reported (Goldblum & Terlikowski, 1912 ▶), although they may not be isostructural with the Sr-bearing analogue (Hennings *et al.*, 2014*c* ▶) due to the large differences in cation radius.

The very large Sr^2+^ cation (*r*_ionic_ = 1.31 Å) in Sr(ClO_4_)_2_·9H_2_O is ninefold coordinated by seven water molecules and two perchlorate O atoms [in a similar fashion to Th(SO_4_)_2_·9H_2_O], leaving two interstitial lattice water molecules. This arrangement results in by far the largest and most intricate polymeric water structure in any of these four groups of 9-hydrates, consisting of a folded (H_2_O)_8_ ring in the *ab* plane linked by the ninth H_2_O to form infinite chains, (H_2_O)_∞_, extending along the *c*-axis. The Ca^2+^ ion (*r*_ionic_ = 1.12 Å) in CaBr_2_·9H_2_O is in eightfold coordination to water, leaving a single interstitial H_2_O. The water molecules in this compound form a branched octamer chain and isolated monomers. Finally, the Mg^2+^ ion (*r*_ionic_ = 0.72 Å) in MgBr_2_·9H_2_O and MgI_2_·9H_2_O exhibits sixfold coordination to water leaving three interstitial lattice water molecules. In this compound, which is arguably the most closely related to MgSeO_4_·9H_2_O, one finds a branched (H_2_O)_9_ chain similar, but not identical, to that in Al(Br)_3_·9H_2_O.

Although substances with a *divalent* cation-to-water ratio of 1:9 are not so exotic (even if there are apparently a number with unknown structures), MgSeO_4_·9H_2_O is quite unusual in being the only example we know of with a cation:*(oxy)anion*:water ratio of 1:1:9. Since it is apparent from the study of other enneahydrates in the four groups detailed above that the anions exercise a degree of control over the polymerization of the water network (at least in the solid state) we should not necessarily expect to find similar polymeric structures in MgSeO_4_·9H_2_O to those already described.

## 5. Summary and conclusion   

We have completed the structure of MgSeO_4_·9H_2_O using neutron single-crystal diffraction methods, confirming the hydrogen-atom positions that had previously been estimated from the heavy-atom structure obtained by X-ray powder diffraction. The degree of agreement with the earlier powder study is a useful and satisfying validation of the methods employed in that work. Moreover, the *ab initio* calculations reproduce the majority of the salient features to high accuracy. This complete structural analysis allows us to understand a range of other observed features, from the crystal morphology through to the anisotropy of the thermal expansion and the frequency of certain vibrational features.

After a thorough comparison of all compounds likely to share structural similarities, we find that MgSeO_4_·9H_2_O is, for now, unique in having a cation-to-anion-to-water ratio of 1:1:9 and in being built around a 12-membered water polymer consisting largely of pentagonal rings.

The stability of the 9- and 11-hydrates with respect to one another and to the previously known heptahydrate remain to be confirmed by detailed study of the binary phase diagram at low temperature. Nonetheless, we have made some observations that suggest that the 9-hydrate is the stable phase in aqueous solution at ∼ 269 K (and is perhaps the stable phase co-existing with ice at the eutectic), whereas the heptahydrate seems likely to be metastable (*i.e.* its solubility is greater than the 9-hydrate) at or around 269 K. Upon warming, MgSeO_4_·11H_2_O transforms to the 9-hydrate; crystals of MgSeO_4_·7H_2_O stored in a freezer at 253 K for 2 weeks were discovered to have transformed entirely to the 9-hydrate; crystals of MgSeO_4_·9H_2_O stored in the same freezer for 5 months were found to be unchanged. Our work provides the means to identify MgSeO_4_·9H_2_O crystals on the basis of their morphology, crystal structure and vibrational spectrum, which will aid in a re-evaluation of the binary phase diagram.

## Supplementary Material

Crystal structure: contains datablock(s) I, II, III, IV. DOI: 10.1107/S2052520615006824/eb5038sup1.cif

Structure factors: contains datablock(s) MgSeO4_9H2O_5K_3D_SXD. DOI: 10.1107/S2052520615006824/eb5038Isup2.hkl

Structure factors: contains datablock(s) MgSeO4_9H2O_100K_3D_SXD. DOI: 10.1107/S2052520615006824/eb5038IIsup3.hkl

Structure factors: contains datablock(s) MgSeO4_9H2O_175K_3D_SXD. DOI: 10.1107/S2052520615006824/eb5038IIIsup4.hkl

Structure factors: contains datablock(s) MgSeO4_9H2O_250K_3D_SXD. DOI: 10.1107/S2052520615006824/eb5038IVsup5.hkl

Supporting figures and tables relating to the sample preparation, ab initio calculations and Raman spectroscopy. DOI: 10.1107/S2052520615006824/eb5038sup6.pdf

CCDC references: 1057947, 1057948, 1057949, 1057950

## Figures and Tables

**Table 1:** Bond lengths () in the polyhedral ions of MgSeO_4_9H_2_O as a function of temperature

	*Ab initio*	5K	100K	175K	250K
SeO1†	1.6621	1.631(3)	1.628(4)	1.626(6)	1.625(6)
SeO2	1.6782	1.640(4)	1.638(5)	1.636(7)	1.635(6)
SeO3	1.6770	1.643(3)	1.639(4)	1.640(5)	1.637(8)
SeO4	1.6811	1.643(3)	1.642(4)	1.639(6)	1.636(6)
**Mean SeO**	**1.6746**	**1.639**	**1.637**	**1.635**	**1.633**
					
MgO*w*1	2.0874	2.061(4)	2.055(6)	2.057(8)	2.065(9)
MgO*w*2	2.0435	2.033(4)	2.035(6)	2.033(7)	2.032(9)
MgO*w*3	2.0976	2.066(4)	2.067(5)	2.066(7)	2.062(7)
MgO*w*4	2.0863	2.057(4)	2.053(6)	2.057(8)	2.061(9)
MgO*w*5	2.0749	2.054(4)	2.056(5)	2.059(7)	2.055(7)
MgO*w*6‡	2.1550	2.111(4)	2.113(6)	2.112(8)	2.111(8)
**Mean MgO**	**2.0908**	**2.064**	**2.063**	**2.064**	**2.064**

† The selenate oxygen accepts two hydrogen bonds (the others each accept three).

‡ The Mg-coordinated water that acts as a hydrogen-bond acceptor.

**Table 2:** Bond lengths () in the polyhedral ions of MgSeO_4_7H_2_O (Fortes Gutmann, 2014 ▶) and MgSeO_4_6H_2_O (Kolitsch, 2002 ▶)

MgSeO_4_7H_2_O		MgSeO_4_6H_2_O	
10K, neutron		293K, X-ray	
SeO1†	1.630(6)	SeO1†	1.627(1)
SeO2	1.642(4)	SeO2	1.640(1)
SeO3†	1.631(8)	SeO3	1.644(1)
SeO4	1.661(7)	SeO4	1.645(1)
**Mean SeO**	**1.641**	**Mean SeO**	**1.639**
			
MgO*w*1	2.045(6)	Mg1O*w*5	2.058(1)
MgO*w*2‡	2.104(8)	Mg1O*w*6	2.063(1)
MgO*w*3	2.046(10)	Mg1O*w*7‡	2.090(1)
MgO*w*4	2.037(6)	Mg2O*w*8	2.047(1)
MgO*w*5‡	2.097(8)	Mg2O*w*9	2.049(1)
MgO*w*6	2.058(9)	Mg2O*w*10	2.087(1)
**Mean MgO**	**2.065**	**Mean MgO**	**2.066**

† The selenate oxygen(s) accepts two hydrogen bonds (the others each accept three).

‡ Mg-coordinated water(s) that acts as a hydrogen-bond acceptor.

**Table 3:** Hydrogen-bond geometry (, ) in MgSeO_4_9H_2_O at 5K and from the *ab initio* calculations (in italics)

Hydrogen-bonded contact	OH	HO	OO	OHO
O*w*1H1*a*O3^i^	0.961(7)	1.820(7)	2.761(4)	165.6(8)
	*0.9847*	*1.7841*	*2.7513*	*166.52*
O*w*1H1*b*O2^ii^	0.971(7)	1.865(7)	2.834(4)	175.9(7)
	*0.9848*	*1.8428*	*2.8263*	*176.34*
O*w*2H2*a*O*w*7^iii^	0.970(7)	1.782(7)	2.741(4)	169.3(8)
	*0.9939*	*1.7137*	*2.6976*	*169.79*
O*w*2H2*b*O*w*9^ii^	0.972(8)	1.803(8)	2.764(5)	169.2(6)
	*0.9889*	*1.7641*	*2.7429*	*169.76*
O*w*3H3*a*O4^iv^	0.970(6)	1.826(6)	2.756(4)	159.5(6)
	*0.9853*	*1.8155*	*2.7570*	*158.74*
O*w*3H3*b*O4^i^	0.975(8)	1.789(8)	2.754(5)	169.6(7)
	*0.9911*	*1.7484*	*2.7320*	*171.14*
O*w*4H4*a*O1	0.977(7)	1.761(7)	2.736(4)	175.8(8)
	*0.9888*	*1.7447*	*2.7321*	*176.15*
O*w*4H4*b*O2^v^	0.972(6)	1.845(6)	2.807(4)	170.1(8)
	*0.9850*	*1.8610*	*2.8340*	*168.93*
O*w*5H5*a*O3	0.973(7)	1.823(8)	2.776(4)	166.0(8)
	*0.9827*	*1.8114*	*2.7812*	*168.48*
O*w*5H5*b*O*w*7	0.977(6)	1.786(6)	2.758(4)	173.2(6)
	*0.9962*	*1.7263*	*2.7198*	*174.63*
O*w*6H6*a*O*w*8^vi^	0.978(7)	1.754(7)	2.729(4)	174.9(7)
	*0.9981*	*1.7130*	*2.7069*	*173.42*
O*w*6H6*b*O*w*9^vi^	0.980(6)	1.781(6)	2.748(4)	168.1(7)
	*0.9998*	*1.7120*	*2.6981*	*168.07*
O*w*7H7*a*O1^vii^	0.972(7)	1.797(7)	2.764(5)	172.7(7)
	*0.9891*	*1.7733*	*2.7552*	*171.35*
O*w*7H7*b*O*w*8	0.974(8)	1.836(8)	2.798(5)	168.9(7)
	*0.9946*	*1.7456*	*2.7306*	*169.98*
O*w*8H8*a*O2^viii^	0.974(7)	1.816(7)	2.769(4)	165.1(6)
	*0.9942*	*1.7559*	*2.7340*	*167.07*
O*w*8H8*b*O4^ix^	0.967(7)	1.835(7)	2.797(4)	172.9(8)
	*0.9888*	*1.8015*	*2.7836*	*171.71*
O*w*9H9*a*O3	0.969(7)	1.844(7)	2.794(4)	165.8(7)
	*0.9884*	*1.8133*	*2.7931*	*170.64*
O*w*9H9*b*O*w*6	0.969(8)	1.978(7)	2.923(4)	164.4(7)
	*0.9931*	*1.8275*	*2.8051*	*167.45*

Symmetry codes: (i) 
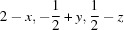
; (ii) 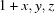; (iii) 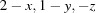; (iv) 
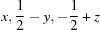
; (v) 
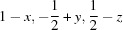
; (vi) 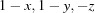; (vii) 
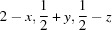
; (viii) 
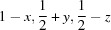
; (ix) 
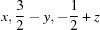
.

**Table 4:** Categorization of the hydrogen bonds in a range of Mg*X*O_4_^2^ hydrates according to donor and acceptor species

Hydration state	Mgwater to sulfate O	Mgwater to free water	Free water to sulfate O	Mgwater to Mgwater	Free water to Mgwater	Free water to free water
Undecahydrate	3	9	8	0	2†	1
Enneahydrate	7	5	4	0	1	1
Heptahydrate	9	2	1	1	1	0
Hexahydrate	11	0	0	1	0	0

† Denotes bifurcated hydrogen bond.

**Table 5:** Properties of the electron density at the BCPs in the hydrogen bonds as determined from the DFT calculations Electron density, (*r*), is reported in e^3^, whereas the Laplacian, ^2^(*r*), and the eigenvalues of the Hessian matrix, _1_, _2_ and _3_, are given in e^5^.

	Fractional coordinates of BCP			Topology of electron density at BCP				
	*x*	*y*	*z*	(*r*)	^2^(*r*)	_1_	_2_	_3_
H1*a*O3	0.2847	0.1364	0.2301	0.2496	2.234	1.405	1.378	5.017
H1*b*O2	0.2819	0.3692	0.2796	0.2231	1.835	1.234	1.205	4.274
H2*a*O*w*7	0.0271	0.3479	0.9647	0.3171	2.584	1.950	1.876	6.411
H2*b*O*w*9	0.2563	0.4432	0.0818	0.2753	2.263	1.635	1.570	5.468
H3*a*O4	0.8054	0.0604	0.9838	0.2375	2.051	1.309	1.262	4.622
H3*b*O4	0.1136	0.0536	0.0789	0.2821	2.316	1.644	1.605	5.564
H4*a*O1	0.6887	0.2628	0.2607	0.2768	2.431	1.634	1.547	5.613
H4*b*O2	0.6737	0.0623	0.1774	0.2151	1.932	1.146	1.119	4.197
H5*a*O3	0.7574	0.5087	0.2277	0.2397	2.094	1.331	1.261	4.686
H5*b*O*w*7	0.9414	0.5952	0.1353	0.2976	2.192	1.869	1.824	5.884
H6*a*O*w*8	0.4703	0.2339	0.9914	0.3124	2.253	1.952	1.930	6.135
H6*b*O*w*9	0.6104	0.4293	0.9540	0.3151	2.250	1.999	1.893	6.142
H7*a*O1	0.1714	0.7548	0.1438	0.2558	2.160	1.486	1.419	5.065
H7*b*O*w*8	0.7980	0.7751	0.0660	0.2921	2.111	1.769	1.745	5.624
H8*a*O2	0.6373	0.9085	0.1111	0.2792	2.285	1.625	1.568	5.478
H8*b*O4	0.6969	0.9288	0.9689	0.2387	1.981	1.359	1.332	4.672
H9*a*O3	0.5320	0.5186	0.1885	0.2377	2.023	1.307	1.285	4.615
H9*b*O*w*6	0.5414	0.4006	0.0603	0.2451	1.769	1.401	1.379	4.548

**Table 6:** Energetic properties of the hydrogen bonds The local kinetic energy density, G(*r*), the local potential energy density, *V*(*r*), and the total energy density, *H*(*r*), at the BCP are all given in atomic units.† The hydrogen-bond energies, *E*_HB_, are in units of kJmol^1^.

	*G*(*r*)	*V*(*r*)	*H*(*r*)	*E*_HB_ [eq. (4)]	*E*_HB_ (corr)	*E*_HB_ [eq. (5)]	*E*_HB_ mean
H1*a*O3	0.02724	0.03130	0.00406	41.09	35.68	30.68	33.18
H1*b*O2	0.02247	0.02590	0.00343	33.99	32.39	25.31	28.85
H2*a*O*w*7	0.03544	0.04407	0.00863	57.85	43.48	39.91	41.70
H2*b*O*w*9	0.02953	0.03559	0.00606	46.73	38.31	33.27	35.79
H3*a*O4	0.02504	0.02880	0.00376	37.81	34.16	28.20	31.18
H3*b*O4	0.03047	0.03692	0.00645	48.47	39.12	34.32	36.72
H4*a*O1	0.03082	0.03643	0.00560	47.82	38.81	34.71	36.76
H4*b*O2	0.02256	0.02509	0.00252	32.93	31.89	25.42	28.65
H5*a*O3	0.02550	0.02928	0.00378	38.44	34.46	28.73	31.59
H5*b*O*w*7	0.03096	0.03919	0.00823	51.45	40.51	34.88	37.69
H6*a*O*w*8	0.03272	0.04206	0.00934	55.22	42.26	36.85	39.55
H6*b*O*w*9	0.03294	0.04255	0.00961	55.86	42.55	37.11	39.83
H7*a*O1	0.02722	0.03203	0.00481	42.05	36.13	30.66	33.39
H7*b*O*w*8	0.02991	0.03793	0.00802	49.80	39.74	33.69	36.71
H8*a*O2	0.03002	0.03633	0.00631	47.69	38.75	33.81	36.28
H8*b*O4	0.02464	0.02873	0.00409	37.72	34.12	27.75	30.94
H9*a*O3	0.02486	0.02873	0.00387	37.71	34.12	28.00	31.06
H9*b*O*w*6	0.02367	0.02900	0.00532	38.06	34.28	26.66	30.47
**Mean**							**34.46**

† Conversion factors used: 1a.u. of (*r*) = 6.7483e^3^; 1a.u. of ^2^(*r*) = 24.099e^5^; 1a.u. of energy density = 2625.4729kJmol^1^.

**Table 7:** Band centres, intensities relative to _1_(SeO_4_^2^) and mode assignments for the Raman spectrum of MgSeO_4_9H_2_O at 78 and 259K

	MgSeO_4_9H_2_O at 78 K		MgSeO_4_9H_2_O at 259 K	
	Raman shift (cm^1^)	Relative intensity	Raman shift (cm^1^)	Relative intensity
(MgO)	229.8	1.6	212.1	1.0
	248.0	1.3	237.0	1.4
				
_2_ (SeO_4_)^2^	361.5	14.1	352.5	11.2
				
_4_ (SeO_4_)^2^	410.1	12.1	403.1	7.4
				
(MgO)	433.2	9.8	425.1	7.7
				
_L_ (H_2_O)	543.9	1.1		
_R_ (H_2_O)	580.3	1.1		
	702.7	1.3		
	767.6	2.1		
				
_1_ (SeO_4_)^2^	845.9	100.0	841.3	100.0
				
_3_ (SeO_4_)^2^	866.7	4.6	861.3	6.2
	888.4	7.5	875.5	6.3
	902.3	9.9	893.9	11.8
				
_2_ (H_2_O)	1660.0	0.7	1661.4	0.6
	1687.9	0.4	1687.2	0.3
	1723.2	0.4	1714.3	0.2
				
_1_ (H_2_O)	3178.4	3.0		
_3_ (H_2_O)	3227.4	9.6	3208.5	2.0
	3282.4	15.3	3277.9	7.9
	3344.0	8.9	3351.7	4.5
	3391.0	11.2	3408.8	9.6
	3428.5	11.8	3463.8	5.3
	3467.0	3.9		

**Table 8:** Unit-cell parameters of MgSeO_4_9H_2_O as a function of temperature and in the athermal limit from DFT calculations

	*Ab initio*	5K	100K	175K	250K
*a* ()	7.2801	7.222(2)	7.226(2)	7.239(2)	7.261(2)
*b* ()	10.5052	10.484(3)	10.487(3)	10.497(3)	10.502(2)
*c* ()	17.2561	17.327(4)	17.330(4)	17.349(4)	17.388(4)
()	109.255	109.57(2)	109.58(2)	109.52(2)	109.42(2)
*V* (^3^)	1245.9	1236.1(6)	1237.4(6)	1242.5(6)	1250.4(5)

## Footnotes

1 Although ‘nonahydrate’ has found common usage to refer to 9-hydrates in the scientific literature this term is both inconsistent with the scheme used to name other hydrates and, moreover, is grammatically inaccurate. ‘Nona’ is derived from the Latin ordinal numbers (and thus means ‘ninth’ rather than ‘nine’), whereas all other prefixes employed by crystallographers are derived from the Greek cardinal numbers.
