# Supplementary material for: Structure of magnesium selenate enneahydrate, MgSeO4·9H2O, from 5 to 250 K using neutron time-of-flight Laue diffraction
Source: Acta Crystallogr B Struct Sci Cryst Eng Mater. 2015 May 26;71(Pt 3):313–27. doi: 10.1107/S2052520615006824 (PMC4450603; doi:10.1107/S2052520615006824)
Supplement: Supplementary file 6 [file b-71-00313-sup6.pdf]

Supplementary Information and Figures

Crystal structure of magnesium selenate enneahydrate, $MgSeO_4 \cdot 9H_2O$, from 5 – 250 K using neutron time-of-flight Laue diffraction

A. Dominic Fortes[1,2,*] *Dario Alfè,*[2] *Eduardo R. Hernández,*[3] *and Matthias J. Gutmann*[4]

[1]Department of Earth Sciences, University College London, Gower Street, London WC1E 6BT, U.K.

[2]Department of Earth and Planetary Sciences, Birkbeck, University of London, Malet Street, London WC1E 7HX, U.K.

[3]Instituto de Ciencia de Materiales de Madrid (ICMM-CSIC), Campus de Cantoblanco, 28049 Madrid, Spain

[4] ISIS Facility, Rutherford Appleton Laboratory, Harwell Science and Innovation Campus, Didcot, Oxfordshire OX11 0QX, UK

*Corresponding author email: andrew.fortes@ucl.ac.uk

CONTENTS

1. Characterization of $MgSeO_4 \cdot 6H_2O$ (p. 2)

2. *Ab initio* atomic coordinates $MgSeO_4 \cdot 9H_2O$ (p. 5)

3. Raman spectroscopic data (p. 7)

1. Characterization of $MgSeO_4{\cdot}6H_2O$

Since we report a hitherto unknown hydration state of a compound that had to be synthesised from scratch, we wish to ensure absolute transparency with regard to our characterization of the starting materials. In particular, it is important to show that the crystalline material produced by reaction of MgO with aqueous H_2SeO_4 and subsequent evaporation was indeed $MgSeO_4{\cdot}6H_2O$, a compound that has previously been characterized by X-ray single-crystal diffraction methods.

A single crystal of approximate dimensions 5 x 5 x 2 mm (Figure S1) was powdered and back-loaded into a standard spinner sample holder. This powder specimen was measured in the range 5 – 150° 2θ over a period of 21 hr using a PANalytical X'Pert Pro powder diffractometer (Ge monochromated radiation, λ = Co $K\alpha_1$ = 1.788996 Å). The data were fitted by the Rietveld method in GSAS/Expgui to yield the unit-cell parameters listed in the first column of Table S1.

The unit-cell parameters are in very good agreement with Kolitsch (2002), the volume being 5.1 % larger than the isotypic sulfate crystal (column 3, Table S1). There are no discernible peaks from any accessory phase in the powder diffraction data (Figures S2 and S3); in other words, the material used to prepare the enneahydrate described in the main body of this work was prepared from phase-pure $MgSeO_4{\cdot}6H_2O$.

Supplementary Figure S1

Photograph of a representative single crystal of $MgSeO_4{\cdot}6H_2O$ (left) and an indexed drawing – prepared using WinXMorph – that shows the faces present (right). The crystal morphology agrees well with that described by Von Groth (1908).

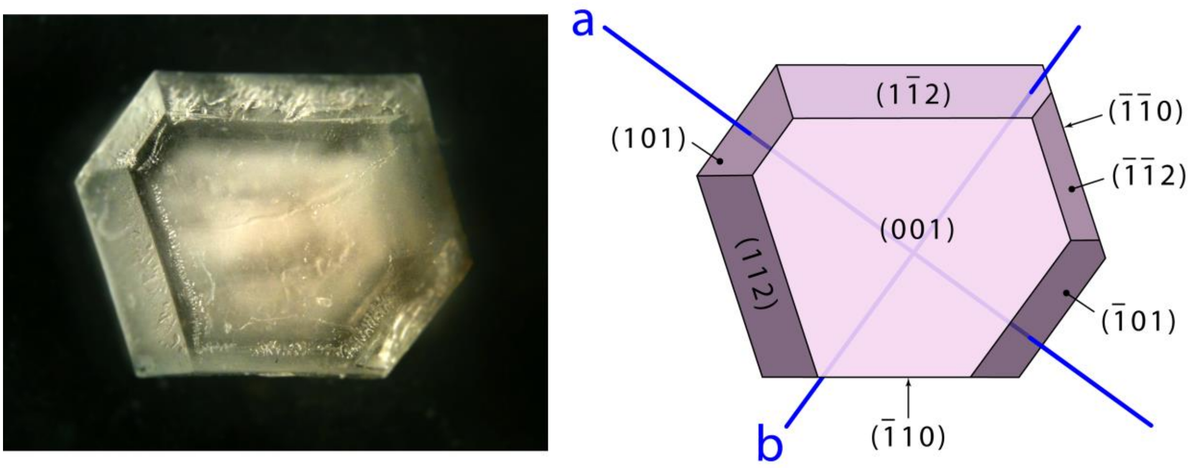

Supplementary Information and Figures

Supplementary Table S1

Unit-cell parameters of $MgSeO_4 \cdot 6H_2O$ compared with the isotypic crystal $MgSO_4 \cdot 6H_2O$.

	$MgSeO_4 \cdot 6H_2O$		$MgSO_4 \cdot 6H_2O$	
	**This work** 298 K	Kolitsch (2002) 293 K	Zalkin *et al*. (1964) "room T"	Batsanov (2000) 120 K
a (Å)	10.21875(9)	10.224(1)	10.110(5)	9.975(2)
b (Å)	7.36537(7)	7.370(1)	7.212(4)	7.186(2)
c (Å)	24.8538(3)	24.866(2)	24.41(1)	24.267(6)
β (°)	98.4087(3)	98.41(1)	98.30(5)	98.78(1)
V (Å)	1850.50(5)	1853.5(3)	1761(1)	1719.1(7)

Supplementary Figure S2

X-ray diffraction pattern of powdered single crystals of $MgSeO_4 \cdot 6H_2O$, measured at room temperature. The red points report the measured data, the green line represents a Rietveld refinement and the pink line underneath is a difference profile. Note the use of a square root intensity scale in order to emphasise weaker peaks.

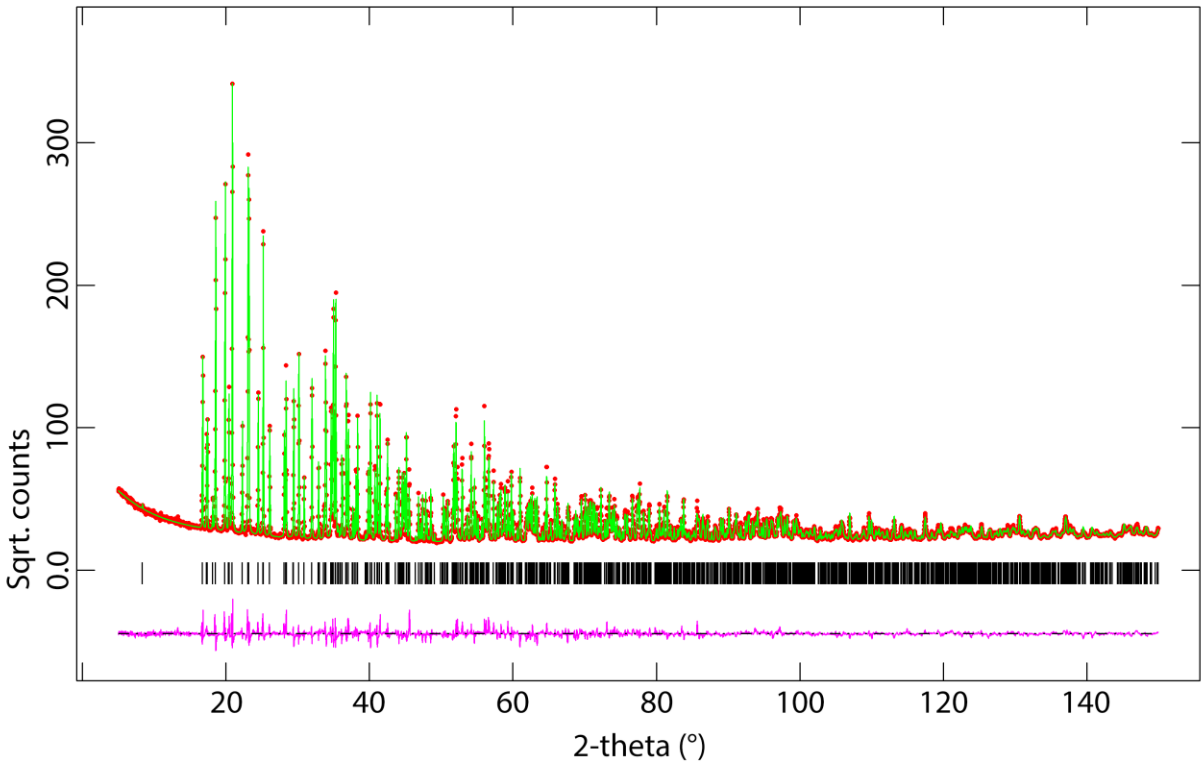

Supplementary Figure S3

Expanded portion of Figure S1, showing the low 2θ range where peaks are well dispersed. This shows very clearly that there are no accessory phases present; the use of a square root intensity scale is designed to emphasise even the weakest of possible parasitic peaks.

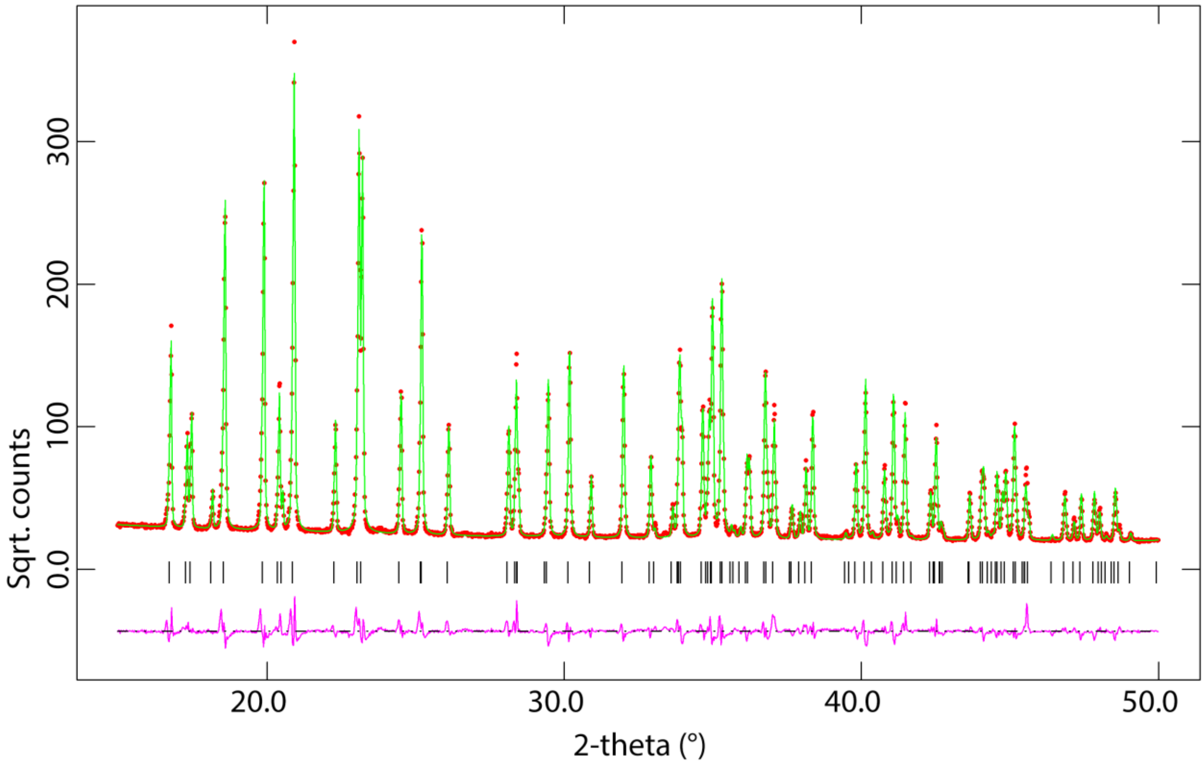

References

Batsanov, A. S. (2000) *Acta Crystallogr. C* **56**, e230–e231.

Kolitsch, U. (2002) *Acta Crystallogr. E* **58**, i3–i5.

Von Groth, P. (1908) *Chemische Krystallographie*, vol. 2, p423.

Zalkin, A., Ruben, H. & Templeton, D. H. (1964). *Acta Crystallgr.* **17**, 235–240.

2. *Ab initio* atomic coordinates $MgSeO_4{\cdot}9H_2O$

Supplementary Table S2

Unit-cell parameters and fractional atomic coordinates of $MgSeO_4{\cdot}9H_2O$ as determined by *ab initio* DFT calculations at zero pressure and temperature.

Space-group $P2_1/c$<br>a = 7.280131 Å<br>b = 10.505192 Å<br>c = 17.256095 Å<br>β = 109.254973°			
Atom	*x*	*y*	*z*
Se	0.60747	0.44527	0.33233
O1	0.67257	0.29611	0.32243
O2	0.37347	0.44991	0.32742
O3	0.63624	0.53669	0.25750
O4	0.74514	0.50394	0.42365
Mg	0.89995	0.29687	0.12261
Ow1	0.15705	0.26082	0.21893
Ow2	0.04907	0.38100	0.05462
Ow3	0.91367	0.11704	0.07198
Ow4	0.72000	0.21251	0.18029
Ow5	0.89739	0.47269	0.17704
Ow6	0.63033	0.33646	0.02608
Ow7	0.97380	0.68180	0.09987
Ow8	0.65789	0.83569	0.03513
Ow9	0.40978	0.49141	0.09444
H9B	0.49724	0.43224	0.07832
H9A	0.47837	0.51296	0.15259
H8B	0.67563	0.89352	0.99295
H8A	0.64744	0.88902	0.08110
H7B	0.86555	0.74440	0.08148
H7A	0.09374	0.73065	0.12750
H6B	0.62364	0.39170	0.97804
H6A	0.53146	0.26807	0.00449
H5B	0.92635	0.55222	0.15181
H5A	0.82004	0.49640	0.21194
H4B	0.70536	0.11928	0.17998
H4A	0.69705	0.24382	0.23036
H3B	0.04319	0.07965	0.07905
H3A	0.83635	0.09329	0.01537

H2B	0.17952	0.42044	0.07559
H2A	0.02707	0.36376	0.99561
H1B	0.22925	0.32942	0.25515
H1A	0.24611	0.18808	0.22522

3. Raman spectroscopic data

Supplementary Table S3

Raman spectroscopic data acquired from single crystals of $MgSeO_4$ hydrates at various temperatures, and from powdered β-$MgSeO_4$. Intensities are in arbitrary units.

Raman shift (cm^{-1})	$MgSeO_4·9H_2O$ 259 K	$MgSeO_4·9H_2O$ 78 K	$MgSeO_4·6H_2O$ Room-T	β-$MgSeO_4$ Room-T
171.23	583.97	345.78	50.00	1914.10
174.09	655.98	457.21	16.61	1520.77
176.95	661.60	357.08	23.50	903.73
179.81	678.98	317.88	74.58	493.05
182.67	725.39	314.56	81.46	318.34
185.53	675.86	155.08	130.40	221.53
188.39	646.44	268.90	241.18	133.95
191.24	629.14	229.32	303.41	113.73
194.09	677.16	347.76	422.80	72.69
196.95	764.19	410.56	510.53	63.38
199.80	836.03	391.41	609.69	100.00
202.65	919.79	193.73	674.74	210.75
205.50	1046.36	463.20	831.49	343.39
208.35	1128.74	537.64	943.00	488.57
211.20	1224.28	600.75	1059.77	738.59
214.05	1255.29	580.90	1127.31	971.95
216.89	1252.30	769.45	1201.09	1241.55
219.74	1190.43	958.86	1247.05	1541.41
222.58	1191.47	1066.59	1219.99	1803.05
225.42	1151.46	1209.74	1255.89	1831.54
228.27	1167.82	1086.01	1247.18	1674.55
231.11	1215.24	1070.53	1256.23	1296.05
233.95	1196.11	1179.31	1272.45	871.44
236.79	1207.59	1156.12	1259.17	573.29
239.62	1160.47	1189.08	1269.16	355.94
242.46	1031.47	1141.93	1266.16	237.55
245.30	887.27	1203.18	1261.09	165.97
248.13	778.22	1249.38	1227.90	129.37
250.97	666.59	1162.24	1183.08	136.31
253.80	550.70	852.38	1060.20	103.58
256.63	454.62	532.97	904.42	129.94
259.46	388.75	440.79	753.73	169.62
262.29	299.43	452.22	623.66	206.34
265.12	265.16	542.68	528.22	266.07

267.95	232.39	392.33	424.74	291.00
270.77	189.33	475.59	409.03	381.62
273.60	185.07	263.47	358.43	470.19
276.43	137.81	233.10	362.11	557.78
279.25	119.20	283.52	376.91	606.28
282.07	53.39	353.30	406.53	648.08
284.89	79.63	353.14	393.68	554.86
287.71	101.82	299.58	389.61	403.39
290.53	170.41	198.60	398.46	277.22
293.35	219.25	231.43	448.54	169.54
296.17	287.93	296.21	464.54	119.06
298.99	404.72	161.25	473.63	109.53
301.80	431.95	406.86	550.36	148.24
304.62	524.28	448.24	607.82	212.44
307.43	563.20	507.08	666.99	266.02
310.25	641.27	666.16	760.70	370.70
313.06	777.19	833.65	847.35	550.33
315.87	860.35	951.65	973.18	770.36
318.68	1034.45	931.48	1088.36	1108.85
321.49	1253.15	826.94	1243.14	1566.46
324.29	1513.39	836.33	1438.71	2169.60
327.10	1880.58	957.76	1668.09	2865.81
329.91	2286.96	1106.02	1928.66	3739.49
332.71	2713.88	1403.77	2231.29	4615.63
335.52	3199.55	2033.40	2599.19	5446.50
338.32	3724.05	2599.92	2950.66	6083.83
341.12	4369.90	3125.82	3273.35	6284.87
343.92	4921.34	3619.83	3622.09	5808.76
346.72	5449.02	3997.43	3851.85	4739.87
349.52	5801.58	4759.12	4077.96	3827.80
352.32	6131.84	5697.56	4126.72	3435.01
355.12	6269.74	6211.66	4111.37	3667.55
357.91	6326.92	6663.29	4097.68	4362.29
360.71	6163.60	6824.15	4099.35	5424.86
363.50	5638.16	6654.50	4089.03	6695.79
366.29	4897.21	6657.99	4101.26	8065.73
369.08	4051.84	6732.58	4073.64	9418.48
371.88	3267.96	6551.44	4007.04	10169.88
374.67	2718.22	5328.91	3772.79	9427.61
377.45	2362.56	3747.34	3482.36	7302.18
380.24	2164.54	2673.21	3175.03	5047.17
383.03	2121.76	2239.33	2886.65	3877.50
385.82	2253.96	2080.51	2632.62	3585.96
388.60	2552.69	2165.33	2499.95	3920.74

391.38	3027.00	2400.17	2419.63	4812.13
394.17	3651.65	3088.76	2488.71	6297.30
396.95	4256.08	3789.03	2608.88	8453.31
399.73	4962.55	4348.75	2743.14	11415.13
402.51	5514.74	5163.25	2967.60	15147.63
405.29	5906.37	5902.93	3207.85	19303.81
408.07	5823.08	6819.13	3457.68	23177.67
410.84	5393.57	7462.74	3674.13	25243.94
413.62	4995.79	7268.85	3878.35	25655.29
416.40	4751.74	5814.86	4044.98	25101.84
419.17	4809.72	4721.54	4181.07	21270.86
421.94	4810.37	4643.56	4327.14	15348.44
424.72	4690.81	5134.80	4419.30	11327.97
427.49	4501.03	5628.43	4479.40	9112.99
430.26	4005.02	5795.08	4484.50	7373.34
433.03	3421.74	5874.70	4481.65	5452.92
435.79	2761.74	5564.95	4330.33	3635.54
438.56	2211.56	5110.70	4196.13	2422.15
441.33	1803.76	3941.90	3940.74	1648.71
444.09	1489.04	2778.86	3690.81	1207.93
446.86	1208.39	2220.32	3382.11	912.20
449.62	1014.97	1792.29	3052.34	694.24
452.38	867.57	1723.95	2757.47	530.80
455.14	710.10	1520.73	2443.38	419.54
457.90	606.55	1307.64	2141.10	321.60
460.66	519.07	1130.92	1847.53	280.90
463.42	448.17	1032.68	1588.56	185.99
466.18	414.74	1074.65	1395.41	178.52
468.94	370.63	1088.98	1209.59	141.26
471.69	302.90	1075.05	1039.17	125.70
474.44	314.83	1098.18	923.12	122.20
477.20	222.19	1042.84	785.22	142.41
479.95	255.22	791.44	654.29	202.85
482.70	213.76	863.21	595.46	256.96
485.45	203.08	792.91	521.07	367.85
488.20	196.72	756.12	453.29	479.06
490.95	181.23	638.42	405.98	616.76
493.70	190.21	758.27	386.71	697.47
496.44	185.70	717.87	334.70	630.38
499.19	170.56	616.88	290.74	507.55
501.94	130.69	540.82	259.12	349.67
504.68	137.79	482.22	233.45	265.06
507.42	128.86	490.09	219.46	236.50
510.16	129.49	324.49	184.61	275.35

512.90	146.94	399.12	176.19	339.51
515.64	115.95	363.71	170.85	420.35
518.38	88.73	309.42	149.67	588.49
521.12	102.43	272.36	141.57	803.40
523.86	96.24	363.94	149.43	1060.53
526.59	123.36	362.42	114.82	1333.18
529.33	105.25	423.91	119.46	1478.59
532.06	86.80	409.86	90.25	1526.54
534.79	89.17	581.16	113.68	1391.95
537.53	101.25	654.36	94.53	1065.60
540.26	99.64	706.57	93.33	748.97
542.99	98.49	727.90	72.10	514.83
545.72	94.01	680.49	59.47	358.01
548.44	75.53	773.55	65.80	276.65
551.17	74.17	575.29	37.58	244.05
553.90	115.32	657.23	65.25	180.65
556.62	52.68	517.06	50.28	202.34
559.35	81.30	378.22	25.77	155.57
562.07	67.73	388.95	44.95	139.09
564.79	67.02	520.59	50.80	131.33
567.51	58.19	443.62	36.17	104.15
570.23	56.92	485.44	43.09	108.69
572.95	35.74	605.38	40.12	123.22
575.67	41.38	685.21	71.34	103.19
578.39	69.50	704.76	43.75	153.87
581.11	64.07	700.90	41.01	107.67
583.82	40.37	693.85	79.99	120.15
586.54	33.82	544.52	51.41	108.83
589.25	54.22	521.44	60.52	117.46
591.96	87.73	409.43	63.17	102.26
594.67	69.99	312.08	47.54	136.26
597.38	44.26	280.72	58.42	119.15
600.09	102.33	363.46	91.80	127.27
602.80	57.02	167.03	56.31	87.14
605.51	83.86	297.59	85.13	140.47
608.22	99.37	370.21	93.71	144.33
610.92	79.68	385.95	118.51	132.61
613.63	85.40	350.34	114.06	129.41
616.33	108.43	378.93	117.53	108.00
619.03	136.77	333.95	109.24	113.43
621.74	98.01	283.21	100.08	168.98
624.44	90.91	388.96	137.35	142.40
627.14	120.77	381.57	118.52	131.96
629.84	103.38	490.65	137.82	129.74

632.53	127.46	366.50	113.68	122.90
635.23	134.68	469.14	135.42	148.55
637.93	171.91	381.57	121.18	133.32
640.62	195.51	531.06	136.60	147.70
643.32	180.90	524.58	158.23	158.32
646.01	163.95	556.83	154.92	152.00
648.70	184.65	545.87	136.27	142.45
651.39	182.81	598.09	147.68	150.36
654.08	204.43	495.38	133.20	133.79
656.77	253.19	474.62	112.17	132.58
659.46	263.81	494.96	123.02	128.98
662.15	237.24	609.69	136.55	147.74
664.84	303.47	625.52	145.36	144.46
667.52	293.30	639.92	138.35	139.77
670.21	322.43	746.45	172.35	123.10
672.89	290.92	778.59	170.83	136.41
675.57	350.06	577.87	134.55	108.18
678.25	400.25	669.06	144.29	135.99
680.94	347.59	706.09	155.04	103.41
683.62	412.29	600.77	132.21	143.51
686.29	390.84	693.75	155.27	133.69
688.97	424.49	850.45	171.47	136.68
691.65	424.14	742.53	150.49	112.15
694.33	458.34	974.25	177.95	159.91
697.00	461.24	1033.17	174.16	155.84
699.67	458.49	1096.88	184.00	154.11
702.35	456.74	1223.64	217.04	171.37
705.02	474.94	1099.65	195.40	173.45
707.69	490.99	1175.56	222.16	213.56
710.36	508.44	1050.95	223.51	159.25
713.03	472.14	985.28	268.29	204.66
715.70	527.24	951.28	254.56	220.60
718.37	534.29	758.62	253.86	264.46
721.03	523.18	806.75	281.45	284.92
723.70	565.53	925.23	316.34	312.53
726.36	483.42	914.65	343.85	308.13
729.03	564.91	873.27	360.83	360.93
731.69	563.24	728.79	365.33	388.91
734.35	564.53	871.09	412.89	431.81
737.01	614.96	875.74	448.74	436.43
739.67	553.69	880.95	478.32	446.10
742.33	616.46	723.70	480.42	466.84
744.99	610.88	966.88	546.48	519.58
747.65	604.65	1069.77	599.74	543.39

750.30	585.96	1097.03	608.02	575.27
752.96	588.37	1239.86	674.53	612.38
755.61	613.17	1182.43	678.42	622.12
758.27	603.82	1481.56	760.38	684.94
760.92	589.72	1424.98	786.96	685.93
763.57	604.55	1555.47	854.63	719.06
766.22	599.74	1589.88	894.33	719.20
768.87	590.57	1475.79	918.15	737.78
771.52	628.29	1443.50	952.70	774.10
774.17	629.60	1275.03	966.57	829.87
776.81	612.31	1176.26	989.53	842.05
779.46	636.66	958.58	998.22	899.94
782.10	713.91	1127.27	1046.92	963.25
784.75	688.65	1063.25	1063.06	1032.62
787.39	756.48	1045.36	1103.41	1111.70
790.03	799.46	847.18	1101.89	1184.56
792.67	873.77	1039.00	1180.19	1266.35
795.31	945.68	1052.97	1213.89	1387.53
797.95	1014.83	1098.68	1262.10	1507.93
800.59	1126.02	984.43	1313.98	1619.81
803.23	1247.01	1133.61	1407.51	1794.12
805.86	1398.48	1115.73	1512.19	2010.92
808.50	1590.30	1373.05	1667.20	2254.67
811.13	1832.36	1578.36	1861.93	2577.47
813.77	2046.70	1861.62	2065.55	3077.82
816.40	2395.14	2055.87	2325.46	3702.95
819.03	2874.72	2502.67	2669.72	4617.11
821.66	3494.59	3315.33	3193.07	6000.58
824.29	4475.89	4073.04	3916.78	7990.66
826.92	6126.79	5107.45	5021.34	10943.76
829.55	9156.68	6638.45	7016.13	14718.02
832.17	15109.75	9390.40	11123.13	18560.36
834.80	24996.37	14486.19	18987.06	21605.59
837.42	37125.82	23405.72	31630.47	22861.40
840.05	46568.26	34223.26	44295.57	22765.74
842.67	49155.64	43581.25	53519.00	23412.91
845.29	41097.66	49125.80	56263.60	27268.88
847.92	26853.46	48295.47	47686.24	34662.08
850.54	15447.40	38617.41	30232.63	43517.60
853.15	9638.94	26516.64	17495.56	49143.50
855.77	7409.41	17536.96	11893.79	47264.52
858.39	6530.11	11981.16	9923.17	37912.19
861.01	6013.90	9398.94	9342.04	26405.40
863.62	5459.48	7937.91	8852.82	17737.72

866.24	4743.39	7032.73	8016.63	12430.57
868.85	4264.24	5736.32	6693.39	9346.93
871.46	4010.88	4767.87	5341.40	7476.08
874.08	4060.90	4136.15	4173.54	6390.79
876.69	4235.71	3929.24	3337.63	5670.16
879.30	4543.25	4050.97	2763.26	5188.17
881.91	4826.28	4289.98	2434.99	4900.31
884.51	5050.40	4632.81	2280.69	4739.05
887.12	5271.84	5088.60	2289.19	4776.36
889.73	5545.37	5441.99	2412.57	4850.29
892.33	5784.09	5845.40	2779.41	5172.32
894.94	5908.74	6490.29	3521.23	5685.11
897.54	5465.98	6999.41	4792.00	6443.60
900.14	4514.70	7348.33	6785.28	7362.96
902.75	3322.89	7004.13	8994.03	8416.27
905.35	2236.23	6229.47	10814.28	10060.22
907.95	1509.00	4815.41	11293.43	12729.10
910.55	1002.20	3186.78	10210.22	17207.84
913.14	714.29	1886.82	7521.83	24711.07
915.74	522.20	1202.91	4736.20	34818.00
918.34	441.30	822.27	2893.65	43538.44
920.93	391.33	621.49	1856.44	45794.72
923.53	316.24	572.40	1297.69	39000.38
926.12	307.04	541.93	964.39	26791.68
928.71	251.41	474.58	764.41	16296.89
931.30	268.17	509.79	628.71	10028.31
933.89	215.41	402.35	515.99	6709.59
936.48	236.38	386.59	454.23	4929.22
939.07	218.43	427.10	399.61	3901.95
941.66	176.96	356.85	343.48	3327.13
944.25	156.42	345.04	308.83	3223.12
946.83	156.16	399.21	320.23	3480.09
949.42	108.93	319.31	253.48	4333.82
952.00	138.09	375.51	239.95	5822.53
954.58	142.12	251.87	209.00	7460.29
957.17	109.12	246.72	195.26	8340.26
959.75	124.31	242.27	190.01	7880.70
962.33	143.33	237.98	165.27	6305.04
964.91	132.33	289.65	172.35	4661.72
967.49	143.45	243.63	148.78	3683.08
970.06	155.36	221.76	136.55	3184.27
972.64	118.59	160.02	124.05	2900.56
975.22	144.05	366.27	164.99	2486.48
977.79	136.24	217.00	116.04	1981.97

980.36	160.31	333.07	125.04	1485.70
982.94	186.90	387.28	142.06	1092.42
985.51	175.62	268.40	116.80	849.56
988.08	144.47	347.55	115.94	717.87
990.65	131.15	328.50	115.64	559.16
993.22	97.05	364.96	111.98	507.28
995.79	99.88	248.47	82.11	435.53
998.36	72.53	326.53	108.05	391.50
1000.92	44.32	184.54	76.03	382.57
1003.49	83.02	131.96	59.06	352.33
1006.05	97.86	125.24	62.04	341.65
1008.62	99.81	193.70	72.77	292.55
1011.18	70.80	324.62	81.28	250.65
1013.74	108.31	200.98	67.47	216.57
1016.30	84.55	196.22	55.87	231.96
1018.86	83.01	175.50	56.38	195.50
1021.42	90.59	304.66	73.31	181.42
1023.98	69.25	243.89	59.09	154.54
1026.54	81.23	125.17	43.27	160.27
1029.09	61.14	247.39	59.54	145.65
1031.65	85.07	244.82	52.45	143.50
1034.21	61.27	298.33	71.18	131.30
1036.76	43.02	205.70	46.59	128.95
1039.31	67.34	312.58	62.74	106.36
1041.86	31.10	330.22	74.97	111.89
1044.41	66.74	327.69	55.58	105.45
1046.96	56.86	118.19	27.67	117.27
1049.51	94.93	323.84	77.20	104.54
1052.06	66.14	328.84	55.61	101.45
1054.61	35.54	291.75	64.20	109.24
1057.16	84.24	193.44	37.74	126.99
1059.70	66.19	196.39	40.26	107.44
1062.25	54.34	275.49	46.69	123.48
1064.79	65.50	283.33	65.81	103.56
1067.33	53.76	345.99	62.97	113.85
1069.87	76.37	287.63	44.91	130.95
1072.42	60.04	333.30	60.90	123.55
1074.96	69.58	231.89	40.58	110.79
1077.49	87.49	291.34	45.84	115.33
1080.03	56.95	331.89	52.52	129.20
1082.57	106.03	232.90	41.35	118.96
1085.11	49.08	320.36	60.13	153.57
1087.64	44.56	224.03	37.13	141.53
1090.18	51.91	226.44	31.65	154.99

1092.71	71.94	324.57	41.50	121.57
1095.24	86.15	337.27	81.79	132.90
1097.78	82.87	344.63	61.84	137.66
1100.31	81.49	360.03	56.65	115.56
1102.84	55.39	234.32	34.38	137.26
1105.37	54.53	297.57	47.93	126.68
1107.89	74.67	307.18	47.27	110.45
1110.42	44.28	222.60	33.97	93.25
1112.95	56.83	358.71	69.60	125.48
1115.47	87.49	332.69	49.78	116.78
1118.00	89.36	346.35	54.62	142.45
1120.52	79.14	287.05	43.09	112.26
1123.04	43.99	315.63	59.05	148.36
1125.57	71.27	318.66	55.74	129.91
1128.09	67.14	177.07	32.89	139.19
1130.61	105.83	305.60	43.60	154.63
1133.13	61.06	292.28	46.24	123.15
1135.65	51.01	368.16	57.84	140.85
1138.16	93.54	279.23	34.98	134.33
1140.68	45.23	350.53	61.07	91.79
1143.20	64.90	231.38	41.53	64.69
1145.71	31.30	215.06	36.79	115.79
1148.22	65.56	152.73	19.35	115.59
1150.74	43.83	291.07	46.31	121.27
1153.25	60.98	304.07	47.63	141.31
1155.76	62.89	309.81	68.62	147.47
1158.27	67.71	220.66	33.27	143.55
1160.78	63.85	328.26	57.50	123.57
1163.29	37.84	346.03	67.22	98.68
1165.80	69.19	311.07	69.65	153.31
1168.30	106.01	305.85	59.88	124.63
1170.81	54.82	148.57	35.38	117.96
1173.31	51.00	323.94	76.58	92.38
1175.82	49.32	322.46	58.49	109.71
1178.32	51.15	208.58	40.33	95.26
1180.82	70.27	278.09	53.98	133.45
1183.32	71.10	315.41	65.41	121.48
1185.82	78.87	322.02	106.80	115.36
1188.32	79.29	133.52	29.97	95.45
1190.82	92.45	147.24	35.68	127.49
1193.32	70.01	134.01	33.56	87.00
1195.82	15.00	319.55	69.55	95.07
1198.31	56.74	200.77	40.85	108.05
1200.81	36.77	113.60	22.59	136.22

1203.30	73.93	322.76	74.04	94.50
1205.79	73.29	183.69	41.36	151.22
1208.29	85.81	262.23	53.52	94.39
1210.78	87.33	194.86	50.00	127.54
1213.27	64.18	115.70	39.48	133.69
1215.76	65.06	293.81	79.16	109.69
1218.25	39.37	226.62	63.09	131.84
1220.74	98.75	246.25	84.46	100.25
1223.22	66.62	144.91	50.90	125.71
1225.71	60.67	207.93	65.31	121.76
1228.19	84.25	258.10	71.80	130.76
1230.68	57.02	328.38	81.12	122.47
1233.16	86.53	109.31	38.52	110.96
1235.64	67.04	228.40	68.03	130.37
1238.13	48.59	178.66	56.11	131.22
1240.61	60.69	110.77	41.39	105.14
1243.09	78.10	103.33	39.45	111.88
1245.56	79.98	204.28	58.54	116.51
1248.04	31.95	251.04	63.07	101.85
1250.52	39.34	295.90	68.54	92.68
1253.00	89.20	293.78	65.45	125.08
1255.47	59.48	165.30	43.24	132.98
1257.95	31.48	280.09	54.12	127.59
1260.42	51.17	241.23	59.51	124.78
1262.89	98.38	102.81	32.57	99.74
1265.36	52.93	189.92	47.27	123.27
1267.84	57.90	253.90	80.57	100.07
1270.31	89.53	237.37	50.38	91.96
1272.77	72.10	236.09	51.95	82.89
1275.24	44.71	167.91	43.66	83.68
1277.71	66.47	228.14	54.08	102.22
1280.18	62.73	169.75	43.26	147.07
1282.64	70.10	219.82	52.29	108.51
1285.11	81.18	212.63	44.14	114.58
1287.57	46.07	316.68	61.44	133.66
1290.03	74.53	287.47	59.87	150.06
1292.50	77.45	247.62	48.13	135.56
1294.96	71.64	307.52	69.20	105.57
1297.42	55.81	262.30	51.17	143.28
1299.88	89.76	171.77	50.71	139.59
1302.34	38.53	239.68	49.08	133.16
1304.79	39.85	263.48	45.32	137.48
1307.25	76.74	181.25	44.90	116.17
1309.71	39.48	258.79	48.61	104.95

1312.16	75.06	245.59	52.62	131.77
1314.62	68.34	227.01	52.30	93.59
1317.07	81.06	297.45	82.28	116.45
1319.52	37.99	166.68	41.10	122.92
1321.97	72.32	237.54	51.28	159.42
1324.42	72.10	165.15	42.96	171.30
1326.87	42.07	297.79	64.78	146.97
1329.32	81.43	223.23	53.66	136.46
1331.77	51.48	163.59	44.27	118.75
1334.22	69.20	213.59	48.99	125.43
1336.66	33.61	219.83	49.04	148.47
1339.11	70.08	267.97	64.99	126.58
1341.55	46.37	259.59	60.30	151.12
1344.00	57.17	64.91	28.68	155.20
1346.44	53.43	306.29	75.46	128.60
1348.88	66.64	131.36	43.17	159.20
1351.32	56.71	204.94	47.45	144.03
1353.76	48.81	74.06	32.95	125.82
1356.20	38.86	139.61	45.50	135.94
1358.64	62.79	241.07	58.57	125.58
1361.08	38.61	126.81	35.28	140.05
1363.51	40.94	189.58	54.75	143.12
1365.95	76.16	268.03	61.82	131.27
1368.39	32.14	262.24	53.59	114.89
1370.82	74.68	57.58	33.86	135.94
1373.25	58.33	254.52	74.00	118.98
1375.68	99.34	103.46	44.57	109.59
1378.12	24.60	195.52	43.07	144.32
1380.55	52.01	273.54	70.57	140.08
1382.98	75.70	133.98	44.10	164.61
1385.40	79.24	211.02	48.10	147.73
1387.83	57.45	146.67	37.13	148.09
1390.26	52.05	221.70	57.33	185.54
1392.69	71.07	278.84	62.53	159.00
1395.11	24.31	91.84	28.06	157.87
1397.54	73.52	281.53	61.42	154.31
1399.96	58.54	184.77	41.75	145.00
1402.38	48.77	281.52	75.52	155.99
1404.80	60.00	169.88	52.91	126.99
1407.22	63.93	277.76	60.71	138.42
1409.64	76.46	238.05	56.67	143.10
1412.06	106.85	332.47	66.43	127.75
1414.48	98.68	299.72	67.93	121.84
1416.90	97.27	204.52	55.55	147.40

1419.32	93.07	302.67	77.21	127.13
1421.73	93.13	323.41	103.47	116.50
1424.15	102.59	206.09	63.07	135.46
1426.56	86.76	252.96	58.49	124.64
1428.97	99.00	282.78	86.62	121.73
1431.39	46.85	211.37	59.71	150.04
1433.80	65.86	226.27	63.34	69.27
1436.21	107.69	276.94	73.63	113.19
1438.62	65.28	336.62	127.69	121.65
1441.03	76.20	334.94	82.99	91.22
1443.43	47.83	223.14	74.35	147.85
1445.84	82.13	343.02	101.64	128.12
1448.25	63.76	334.25	114.13	109.23
1450.65	56.11	342.73	122.29	84.37
1453.06	51.74	303.04	134.85	76.79
1455.46	69.69	230.36	115.58	114.82
1457.86	106.77	264.79	127.64	131.13
1460.27	78.93	230.46	127.20	114.65
1462.67	82.32	293.56	131.82	122.90
1465.07	87.94	316.02	145.18	147.81
1467.47	75.49	326.93	141.64	119.90
1469.86	62.73	220.12	122.61	118.30
1472.26	69.41	359.71	132.57	138.97
1474.66	80.47	162.81	107.73	140.05
1477.05	59.22	234.13	116.06	75.91
1479.45	91.01	343.92	153.69	122.17
1481.84	82.29	360.40	151.85	142.96
1484.24	87.11	286.98	141.45	147.61
1486.63	71.07	322.11	158.01	102.13
1489.02	37.79	237.42	142.24	120.58
1491.41	72.54	320.41	150.27	92.39
1493.80	47.60	358.21	159.07	129.52
1496.19	67.55	254.64	118.93	111.75
1498.58	95.10	299.22	120.97	99.55
1500.96	43.81	240.60	98.77	139.57
1503.35	65.62	317.98	97.00	118.16
1505.74	64.03	371.24	98.30	142.34
1508.12	36.99	331.80	91.67	102.96
1510.50	31.47	241.36	72.77	99.92
1512.89	75.95	298.04	75.47	98.30
1515.27	81.59	326.72	78.04	175.14
1517.65	62.99	246.69	64.65	136.70
1520.03	55.40	338.35	75.19	161.71
1522.41	78.67	366.09	95.76	130.19

1524.79	54.36	305.75	70.90	103.77
1527.17	76.56	182.67	44.74	134.77
1529.54	82.03	379.19	91.88	116.29
1531.92	50.27	159.54	47.92	121.36
1534.30	53.82	236.74	58.00	125.23
1536.67	31.50	356.38	70.61	134.64
1539.04	71.15	139.81	29.89	142.40
1541.42	49.07	218.57	40.90	148.71
1543.79	31.96	149.05	38.25	150.39
1546.16	12.71	129.76	30.29	120.00
1548.53	62.44	305.90	59.34	141.82
1550.90	76.09	350.22	73.02	187.02
1553.27	67.76	339.91	68.64	158.81
1555.63	93.80	222.23	45.96	167.90
1558.00	73.86	228.09	47.98	129.71
1560.37	112.14	203.21	45.43	141.72
1562.73	68.23	340.98	54.49	128.11
1565.10	44.95	236.81	48.21	143.49
1567.46	63.93	292.37	58.30	130.52
1569.82	61.23	126.91	40.53	163.21
1572.18	62.60	199.42	53.82	156.75
1574.54	45.34	199.73	51.55	167.77
1576.90	74.09	212.07	48.11	142.52
1579.26	64.17	341.92	90.53	162.18
1581.62	26.86	292.50	84.63	163.44
1583.98	69.11	338.70	70.89	141.79
1586.33	83.53	293.64	68.82	147.22
1588.69	4.47	328.60	77.82	127.47
1591.04	72.43	148.12	42.89	110.58
1593.40	78.90	328.63	76.72	110.49
1595.75	112.19	209.26	53.99	125.37
1598.10	68.84	233.82	59.59	145.23
1600.45	95.16	307.03	69.79	126.50
1602.81	78.84	211.07	61.26	129.10
1605.15	74.14	344.07	83.00	139.60
1607.50	91.85	320.08	71.53	152.60
1609.85	61.78	213.55	62.70	151.06
1612.20	102.02	381.95	110.27	117.93
1614.54	64.18	233.06	74.78	139.05
1616.89	106.25	352.52	93.82	102.57
1619.23	120.48	380.83	117.25	155.87
1621.58	105.88	350.57	89.43	109.99
1623.92	104.29	218.97	74.73	139.21
1626.26	121.81	163.85	62.57	132.24

1628.60	132.65	239.60	78.18	130.87
1630.95	132.75	317.08	88.85	173.12
1633.28	115.91	294.80	90.23	128.38
1635.62	144.48	219.01	80.23	125.93
1637.96	139.46	303.20	98.11	82.63
1640.30	134.76	370.98	110.05	105.55
1642.63	189.12	374.73	132.72	122.76
1644.97	183.09	408.59	139.83	136.66
1647.30	194.92	285.38	119.14	105.20
1649.64	207.41	409.57	152.24	126.91
1651.97	255.37	426.83	183.62	161.95
1654.30	281.68	448.54	126.69	126.88
1656.63	358.05	679.59	170.00	122.85
1658.96	389.19	602.21	135.87	140.13
1661.29	392.53	570.71	134.28	104.57
1663.62	362.39	500.19	156.15	87.86
1665.95	335.85	532.19	169.36	106.08
1668.28	361.97	493.31	172.20	87.80
1670.60	295.15	479.37	190.77	87.87
1672.93	300.64	495.38	169.85	89.75
1675.25	269.69	346.03	148.25	122.39
1677.58	268.70	404.67	166.66	104.15
1679.90	250.47	347.52	150.89	90.90
1682.22	298.34	492.60	177.93	78.82
1684.54	316.32	481.51	207.37	108.84
1686.86	284.56	553.80	191.61	87.01
1689.18	259.46	533.37	190.51	105.73
1691.50	248.92	368.12	137.88	113.56
1693.82	258.38	405.85	137.35	86.16
1696.13	189.95	506.89	140.78	129.04
1698.45	179.03	317.79	101.62	149.39
1700.77	185.51	311.06	101.08	151.98
1703.08	192.35	387.00	117.95	96.58
1705.39	141.59	398.51	110.35	85.57
1707.71	184.99	327.50	88.81	122.46
1710.02	170.10	382.99	109.01	95.92
1712.33	180.86	337.98	81.07	85.10
1714.64	212.53	288.32	63.04	92.41
1716.95	188.20	449.48	90.13	91.01
1719.26	127.28	347.39	66.93	97.29
1721.56	120.66	475.92	119.29	77.07
1723.87	131.25	484.44	72.78	79.75
1726.18	122.49	458.52	88.34	69.19
1728.48	100.54	348.64	68.09	96.15

1730.79	115.19	271.26	47.72	65.08
1733.09	123.69	310.45	79.81	117.79
1735.39	76.40	290.75	64.43	90.28
1737.69	67.51	315.63	67.15	91.21
1740.00	69.33	214.98	53.83	114.59
1742.30	70.24	211.32	57.52	96.93
1744.59	44.51	324.89	91.10	121.43
1746.89	72.48	171.63	45.35	81.04
1749.19	101.31	305.28	72.40	133.98
1751.49	56.59	323.02	83.18	84.74
1753.78	105.32	289.15	66.66	123.04
1756.08	67.25	170.84	35.80	111.93
1758.37	82.68	312.96	65.88	132.47
1760.67	73.22	192.44	42.98	135.72
1762.96	77.96	181.81	34.36	131.77
1765.25	63.29	269.96	70.73	88.59
1767.54	54.23	175.39	45.37	97.89
1769.83	88.02	276.67	64.80	107.11
1772.12	60.66	227.74	42.60	113.67
1774.41	66.50	245.74	40.57	131.57
1776.70	47.25	306.50	58.44	131.27
1778.99	71.14	192.46	29.06	153.43
1781.27	52.18	291.18	77.73	182.13
1783.56	70.27	182.40	32.59	112.51
1785.84	60.86	330.91	54.01	112.27
1788.12	76.15	273.73	37.40	103.65
1790.41	31.74	297.12	61.59	137.44
1792.69	68.93	309.08	52.45	135.39
1794.97	29.87	327.14	64.20	147.68
1797.25	61.75	189.18	26.59	143.54
1799.53	74.89	189.05	31.77	128.55
1801.81	35.32	344.29	60.69	146.29
1804.09	110.05	276.68	41.64	144.22
1806.36	64.28	187.61	23.02	135.69
1808.64	82.06	283.95	53.73	127.79
1810.92	51.99	248.75	34.78	142.07
1813.19	44.86	273.10	75.32	143.81
1815.46	55.14	314.87	40.94	130.48
1817.74	66.31	250.78	37.68	160.23
1820.01	85.28	298.00	45.42	158.08
1822.28	39.66	259.57	66.23	179.83
1824.55	75.13	234.46	36.84	188.30
1826.82	50.41	292.26	81.62	162.78
1829.09	71.28	295.45	47.92	155.19

1831.36	49.16	315.80	47.30	105.25
1833.62	36.64	276.12	66.97	150.42
1835.89	91.22	245.19	35.34	117.47
1838.16	73.05	165.07	25.28	144.45
1840.42	32.99	208.84	30.76	158.06
1842.68	53.72	255.82	51.76	154.92
1844.95	70.56	298.24	69.93	161.10
1847.21	45.81	245.69	43.21	130.25
1849.47	55.00	210.46	35.71	146.96
1851.73	72.20	281.89	50.25	160.71
1853.99	51.85	262.58	48.62	186.48
1856.25	28.66	266.33	43.96	127.64
1858.51	96.02	259.35	43.94	154.37
1860.77	26.54	290.46	39.24	150.40
1863.02	62.11	252.81	37.55	154.79
1865.28	45.63	263.91	61.02	135.17
1867.53	42.26	272.56	38.23	135.19
1869.79	29.15	196.95	32.84	124.30
1872.04	56.84	191.95	29.22	161.40
1874.29	91.24	248.42	38.36	100.60
1876.54	14.10	294.93	45.57	100.88
1878.79	65.81	119.04	29.51	88.97
1881.04	79.73	47.47	12.59	115.34
1883.29	46.80	142.11	19.90	148.00
1885.54	60.94	146.75	29.55	127.03
1887.79	64.18	186.29	37.83	111.39
1890.04	73.63	56.87	21.17	155.37
1892.28	76.18	230.31	37.02	121.93
1894.53	47.30	265.60	61.77	146.38
1896.77	83.32	253.59	47.92	106.97
1899.01	49.40	179.44	33.41	71.99
1901.26	57.75	211.58	52.23	110.73
1903.50	88.80	195.39	32.55	85.01
1905.74	12.06	268.10	57.24	140.04
1907.98	21.28	279.80	57.03	130.17
1910.22	61.81	202.21	39.65	143.12
1912.46	56.55	254.45	64.88	111.96
1914.70	60.00	266.60	59.51	118.23
1916.93	88.86	276.38	56.87	122.26
1919.17	72.53	290.40	59.83	85.92
1921.40	40.06	247.78	60.46	103.24
1923.64	105.19	226.40	31.76	96.93
1925.87	68.02	219.73	34.83	75.70
1928.11	56.00	279.16	47.54	114.94

1930.34	37.03	207.50	40.91	105.07
1932.57	72.95	283.76	73.58	115.39
1934.80	87.26	166.80	25.33	106.08
1937.03	37.96	295.72	64.04	116.67
1939.26	56.35	155.53	31.18	112.63
1941.49	75.73	284.72	46.87	101.95
1943.71	84.93	307.36	77.88	131.46
1945.94	61.77	304.04	66.73	93.91
1948.17	57.83	313.00	48.56	145.40
1950.39	89.57	284.59	56.85	136.50
1952.61	78.68	302.09	64.34	131.05
1954.84	95.27	331.54	61.63	104.90
1957.06	103.37	267.12	45.26	77.19
1959.28	51.54	310.84	46.43	87.86
1961.50	71.57	285.84	60.50	133.07
1963.72	61.36	306.60	52.04	109.64
1965.94	118.56	218.33	34.91	119.61
1968.16	61.37	118.56	26.05	152.68
1970.38	78.99	300.86	68.40	125.62
1972.59	56.65	221.86	40.58	129.24
1974.81	56.97	275.40	53.89	159.52
1977.02	65.24	304.55	60.41	99.10
1979.24	48.50	326.50	80.20	127.12
1981.45	65.25	163.78	34.32	92.22
1983.67	77.70	221.49	43.68	101.95
1985.88	70.88	265.47	64.67	109.64
1988.09	94.25	257.24	71.70	91.27
1990.30	72.80	244.39	43.94	106.80
1992.51	86.99	287.19	52.74	100.16
1994.72	54.26	227.13	50.97	91.93
1996.92	46.50	169.03	37.51	88.66
1999.13	59.72	226.81	48.92	98.61
2001.34	68.62	309.70	61.50	88.09
2003.54	79.53	304.11	71.88	103.34
2005.75	86.52	274.74	66.16	71.28
2007.95	69.93	123.20	24.44	116.16
2010.16	108.31	122.06	33.49	120.96
2012.36	25.09	124.38	34.38	96.99
2014.56	74.04	271.39	49.90	145.40
2016.76	84.44	207.11	50.96	139.23
2018.96	76.48	136.11	35.25	128.53
2021.16	54.07	207.34	47.38	128.02
2023.36	83.26	218.84	50.05	107.26
2025.56	104.20	341.64	55.48	139.29

2027.75	65.51	272.58	56.88	142.02
2029.95	92.72	274.88	57.68	135.78
2032.14	72.70	287.76	53.59	162.68
2034.34	74.75	270.91	65.86	115.64
2036.53	80.03	338.60	83.30	135.41
2038.72	63.78	349.38	65.82	117.97
2040.92	70.61	314.89	79.60	120.00
2043.11	74.93	355.91	73.18	168.38
2045.30	79.58	143.53	38.96	198.27
2047.49	57.33	275.95	58.48	116.63
2049.68	38.52	284.70	57.47	144.61
2051.86	66.91	320.48	76.56	137.32
2054.05	63.56	293.11	64.91	117.13
2056.24	70.50	203.16	57.21	122.25
2058.42	72.12	130.10	45.00	135.15
2060.61	98.04	247.56	56.03	179.59
2062.79	75.18	153.27	38.16	141.28
2064.97	58.24	201.72	44.46	128.40
2067.16	97.57	330.50	69.60	135.16
2069.34	48.99	327.69	81.96	141.81
2071.52	79.23	284.99	58.30	130.25
2073.70	43.71	211.57	51.41	122.56
2075.88	43.46	347.37	79.62	131.37
2078.06	72.39	266.54	55.76	124.76
2080.23	65.26	323.55	80.74	149.36
2082.41	82.05	269.66	61.09	129.56
2084.59	75.22	336.20	74.13	143.15
2086.76	59.23	215.41	41.12	142.67
2088.94	58.10	201.76	44.10	118.72
2091.11	78.06	324.87	66.99	98.27
2093.28	78.40	332.29	72.18	121.13
2095.46	55.32	331.64	94.50	119.29
2097.63	33.41	317.68	92.62	135.47
2099.80	75.09	178.09	41.31	117.69
2101.97	61.69	243.79	55.11	121.72
2104.14	54.72	147.07	37.50	134.21
2106.30	62.93	281.42	63.57	110.95
2108.47	53.31	239.31	51.10	100.64
2110.64	68.48	288.64	56.43	103.02
2112.80	59.47	292.39	57.03	91.52
2114.97	40.18	257.25	56.30	124.70
2117.13	65.03	210.98	48.74	107.22
2119.30	56.15	206.72	49.18	108.33
2121.46	49.25	325.63	71.48	93.60

2123.62	78.23	156.89	41.33	48.65
2125.78	72.78	120.15	35.80	89.80
2127.94	53.86	125.31	37.65	100.80
2130.10	84.46	328.52	73.19	63.61
2132.26	74.84	310.23	81.74	113.30
2134.42	74.70	336.37	71.89	99.67
2136.57	48.59	182.60	48.94	94.68
2138.73	47.69	288.14	54.92	106.33
2140.89	52.98	269.08	55.21	88.88
2143.04	49.04	223.01	58.43	96.70
2145.20	75.02	169.22	49.78	92.39
2147.35	69.33	176.56	54.40	115.71
2149.50	51.82	285.30	64.36	106.56
2151.65	90.33	287.12	73.98	94.96
2153.80	58.96	107.45	36.31	128.16
2155.95	54.37	228.17	64.47	123.32
2158.10	63.81	299.64	81.60	121.65
2160.25	61.61	243.21	51.36	138.81
2162.40	54.09	306.21	69.93	147.05
2164.55	58.49	321.33	72.76	87.01
2166.69	85.12	191.47	55.79	123.01
2168.84	16.21	252.46	85.86	93.14
2170.98	83.28	246.49	66.76	117.91
2173.13	80.22	136.45	52.23	109.26
2175.27	55.13	219.00	62.44	108.40
2177.41	77.76	172.74	50.41	124.75
2179.55	56.56	219.31	58.62	75.71
2181.69	64.18	85.87	41.59	100.94
2183.83	87.42	234.93	79.10	112.62
2185.97	46.63	201.05	55.78	99.03
2188.11	75.86	124.94	52.66	59.69
2190.25	59.96	190.25	66.61	87.12
2192.38	36.97	238.08	73.69	111.14
2194.52	82.56	245.08	86.30	60.75
2196.66	64.46	132.60	71.68	86.17
2198.79	35.42	164.15	54.10	88.70
2200.92	19.91	260.41	71.29	131.99
2203.06	50.76	287.32	81.47	108.33
2205.19	79.38	226.20	87.09	122.40
2207.32	45.41	279.90	78.72	159.36
2209.45	84.66	88.43	49.54	112.58
2211.58	52.77	249.44	79.40	70.14
2213.71	48.85	102.16	53.02	124.58
2215.84	55.79	161.84	60.58	95.80

2217.96	60.74	69.55	51.34	89.20
2220.09	27.82	58.00	57.47	140.25
2222.22	55.25	226.53	80.43	104.95
2224.34	80.44	88.83	57.76	109.13
2226.47	59.95	194.15	76.66	94.38
2228.59	77.72	242.28	80.59	127.53
2230.71	53.76	142.31	75.72	114.98
2232.84	91.75	163.29	73.86	96.67
2234.96	41.11	304.68	118.59	101.06
2237.08	68.08	345.49	113.20	134.33
2239.20	86.06	122.30	77.57	127.62
2241.32	20.64	141.77	80.51	140.65
2243.43	48.44	185.02	87.21	98.85
2245.55	88.85	236.92	95.35	124.65
2247.67	33.57	209.31	103.59	133.54
2249.78	52.04	318.35	117.00	181.88
2251.90	60.58	207.83	95.11	190.77
2254.01	68.72	326.05	121.59	161.91
2256.13	33.98	273.83	109.37	181.26
2258.24	56.43	310.81	117.85	203.57
2260.35	73.90	296.05	131.06	205.04
2262.46	35.57	108.50	100.31	175.04
2264.57	65.75	270.32	120.69	169.86
2266.68	65.34	268.58	122.87	125.25
2268.79	75.24	278.33	136.32	178.43
2270.90	80.84	281.04	133.61	191.90
2273.01	28.04	265.22	139.00	192.60
2275.11	76.20	255.48	139.55	184.09
2277.22	48.66	302.14	178.50	139.43
2279.32	53.03	191.59	130.32	190.37
2281.43	80.46	294.49	169.91	112.87
2283.53	65.73	193.49	133.47	124.42
2285.63	67.86	269.25	159.89	131.53
2287.74	87.65	318.77	162.75	148.06
2289.84	54.09	323.28	175.45	139.01
2291.94	42.38	324.22	180.11	113.83
2294.04	69.82	289.72	176.43	107.13
2296.14	75.31	129.93	147.53	99.81
2298.23	51.31	254.60	174.93	109.32
2300.33	84.85	186.91	171.52	84.62
2302.43	51.40	328.70	196.39	118.46
2304.52	89.15	119.58	169.22	93.09
2306.62	55.90	137.39	173.79	99.02
2308.71	62.85	328.86	212.39	111.86

2310.81	82.20	148.12	192.62	105.65
2312.90	22.50	285.41	215.72	101.11
2314.99	62.40	326.24	235.76	130.67
2317.08	69.69	318.67	247.36	108.00
2319.17	65.29	226.20	241.07	93.11
2321.26	33.18	305.36	275.17	68.38
2323.35	78.28	217.10	254.31	86.89
2325.44	114.72	220.35	269.28	80.32
2327.53	74.46	177.74	279.69	173.62
2329.61	135.84	253.96	292.57	154.85
2331.70	153.23	361.44	315.77	116.41
2333.78	123.51	663.22	373.28	111.59
2335.87	77.64	587.55	349.79	124.10
2337.95	50.52	437.14	355.27	94.28
2340.03	104.74	446.63	383.62	78.48
2342.12	58.27	192.15	366.40	76.22
2344.20	46.05	263.64	401.81	78.17
2346.28	103.47	321.32	427.52	123.20
2348.36	50.84	355.53	463.17	161.85
2350.44	75.61	380.89	477.49	119.38
2352.51	78.22	267.50	486.54	160.78
2354.59	107.19	253.13	513.79	146.75
2356.67	89.65	268.66	553.97	157.85
2358.74	68.81	268.21	566.29	163.32
2360.82	104.37	374.78	621.34	119.77
2362.89	77.13	264.60	625.76	137.23
2364.97	93.39	314.96	670.51	126.98
2367.04	98.70	376.13	715.23	152.79
2369.11	86.55	190.32	707.19	155.92
2371.18	66.81	191.32	737.28	141.18
2373.25	52.71	187.70	755.03	120.21
2375.32	86.76	392.17	829.42	101.31
2377.39	60.76	328.50	818.08	100.03
2379.46	70.81	195.84	818.94	128.47
2381.53	66.41	347.07	854.26	143.59
2383.60	87.90	347.79	869.22	117.53
2385.66	85.50	366.13	913.82	84.49
2387.73	107.64	255.91	885.34	127.37
2389.79	65.18	193.39	887.40	105.66
2391.86	94.28	290.35	908.27	135.79
2393.92	75.52	337.69	932.46	130.68
2395.98	102.90	320.36	954.89	115.64
2398.04	55.39	385.39	991.95	75.52
2400.10	69.48	248.63	980.68	81.92

2402.16	99.57	368.40	1035.58	63.74
2404.22	92.45	245.06	1027.11	92.63
2406.28	84.19	386.21	1067.38	82.49
2408.34	63.67	375.76	1084.31	66.02
2410.40	91.30	309.43	1084.25	42.87
2412.45	60.38	240.00	1087.35	47.94
2414.51	85.02	380.37	1136.35	86.73
2416.56	66.59	196.71	1101.92	65.33
2418.62	77.03	305.57	1115.79	102.66
2420.67	70.70	370.89	1134.69	80.10
2422.72	63.78	343.86	1171.49	100.66
2424.77	43.36	230.84	1169.06	94.78
2426.82	49.48	297.73	1239.43	76.98
2428.87	61.41	297.55	1242.87	69.24
2430.92	11.68	158.95	1277.61	72.63
2432.97	70.16	216.18	1335.85	62.73
2435.02	67.98	220.70	1411.42	53.75
2437.07	16.46	306.18	1539.46	81.68
2439.11	29.53	313.17	1609.94	92.63
2441.16	78.15	128.70	1635.38	84.60
2443.20	48.42	151.20	1721.95	118.38
2445.25	62.40	308.63	1834.10	120.44
2447.29	29.72	325.52	1922.87	103.35
2449.33	48.94	145.88	1930.55	111.83
2451.38	70.36	253.03	1987.26	132.29
2453.42	64.48	215.43	2018.24	113.60
2455.46	82.90	128.03	2013.25	84.39
2457.50	85.07	317.16	2065.86	95.38
2459.54	55.29	261.94	2043.34	136.15
2461.57	64.91	279.46	2055.58	160.67
2463.61	86.97	323.38	2044.16	144.76
2465.65	85.39	291.71	2035.08	144.23
2467.68	33.06	208.75	2004.32	134.49
2469.72	75.38	271.31	2034.64	154.59
2471.75	59.75	281.58	2044.95	150.93
2473.79	71.77	317.34	2072.27	162.91
2475.82	51.38	336.09	2109.68	145.64
2477.85	83.30	277.59	2153.53	159.88
2479.88	67.27	142.11	2197.01	152.94
2481.91	81.59	269.86	2260.90	134.96
2483.94	60.25	128.08	2282.91	149.65
2485.97	55.82	212.93	2328.10	202.50
2488.00	71.89	301.10	2381.31	147.17
2490.03	70.76	308.29	2355.63	152.34

2492.06	35.23	343.41	2390.02	161.39
2494.08	115.84	294.10	2305.74	178.79
2496.11	87.06	217.21	2276.75	164.51
2498.13	31.53	164.10	2272.37	157.18
2500.16	59.95	262.82	2308.16	178.23
2502.18	89.22	272.64	2360.35	199.03
2504.20	101.74	314.47	2407.37	187.94
2506.22	14.50	288.46	2538.05	147.62
2508.25	81.28	196.46	2560.21	175.17
2510.27	62.05	315.80	2649.63	170.32
2512.29	72.67	293.51	2683.49	163.04
2514.30	87.09	173.74	2639.61	136.20
2516.32	78.56	300.73	2611.80	172.29
2518.34	74.58	240.98	2515.99	163.94
2520.36	68.50	181.65	2411.91	143.81
2522.37	54.02	264.24	2334.93	135.87
2524.39	54.75	143.76	2236.92	70.71
2526.40	52.17	123.24	2163.81	119.99
2528.41	77.59	157.51	2116.10	129.94
2530.43	70.12	195.24	2086.36	126.91
2532.44	64.79	154.41	2051.39	149.28
2534.45	66.27	227.82	2058.01	103.17
2536.46	50.79	200.82	2056.60	120.96
2538.47	26.32	192.23	2027.73	101.06
2540.48	66.85	261.03	2034.85	130.18
2542.49	59.73	117.95	1982.75	86.15
2544.50	44.26	264.51	1944.54	123.49
2546.50	18.43	173.73	1849.33	114.27
2548.51	66.42	105.43	1744.93	82.62
2550.51	71.14	243.07	1671.99	119.28
2552.52	69.88	254.62	1575.95	86.10
2554.52	42.11	211.48	1463.67	114.93
2556.53	18.14	205.38	1385.80	108.42
2558.53	38.78	278.34	1290.93	109.41
2560.53	50.71	211.40	1204.76	128.91
2562.53	88.00	229.84	1143.39	95.67
2564.53	31.84	219.90	1094.78	108.49
2566.53	71.87	185.71	1037.14	134.32
2568.53	64.91	234.65	1005.21	122.60
2570.53	77.95	269.86	978.20	178.35
2572.52	84.09	239.51	950.60	157.79
2574.52	53.18	192.10	910.36	138.10
2576.52	54.93	136.47	890.02	178.47
2578.51	77.17	262.37	891.05	142.39

2580.51	30.67	278.37	876.75	175.27
2582.50	62.61	208.70	861.51	158.16
2584.49	83.66	149.46	848.24	132.10
2586.48	57.90	242.30	861.24	125.39
2588.48	80.30	181.66	861.30	151.86
2590.47	45.80	230.10	886.13	155.87
2592.46	90.50	308.31	936.03	170.84
2594.45	73.95	319.12	941.40	176.06
2596.43	69.20	192.23	951.83	183.68
2598.42	87.10	114.30	967.29	180.72
2600.41	93.55	291.85	1030.46	161.27
2602.40	103.80	177.53	1045.92	167.62
2604.38	83.30	325.72	1099.52	193.07
2606.37	66.31	219.14	1076.59	173.53
2608.35	55.16	194.60	1060.22	197.59
2610.33	63.81	226.96	1033.12	191.71
2612.32	72.22	268.89	1003.68	168.34
2614.30	65.37	315.12	969.85	195.41
2616.28	66.18	283.96	915.68	129.40
2618.26	40.73	321.25	877.65	142.38
2620.24	87.44	204.58	824.24	131.63
2622.22	65.99	305.71	809.11	100.77
2624.20	4.25	318.07	757.34	141.93
2626.17	57.90	207.50	712.04	142.57
2628.15	65.16	206.79	684.74	174.39
2630.13	110.02	227.34	648.21	134.95
2632.10	25.02	119.55	609.34	118.36
2634.08	53.93	325.17	622.44	154.74
2636.05	106.08	192.00	576.81	115.51
2638.02	60.59	130.07	543.71	96.98
2639.99	78.74	212.18	539.27	99.11
2641.97	62.65	142.04	506.04	137.10
2643.94	85.41	260.30	505.17	125.33
2645.91	52.41	289.66	492.24	144.92
2647.88	103.92	299.04	485.71	106.56
2649.85	47.82	295.00	459.27	104.51
2651.81	10.07	200.89	437.91	135.85
2653.78	76.73	123.91	402.01	110.65
2655.75	39.63	347.98	426.77	118.05
2657.71	69.23	304.21	411.14	78.75
2659.68	83.59	327.07	396.74	83.56
2661.64	71.39	225.65	356.94	105.41
2663.61	88.55	309.73	355.47	117.38
2665.57	68.45	358.76	365.41	133.58

2667.53	51.35	165.73	320.91	80.34
2669.49	63.25	201.28	309.14	85.25
2671.45	29.80	218.27	303.27	99.76
2673.41	68.15	289.80	302.94	95.58
2675.37	70.70	349.77	315.41	113.90
2677.33	61.70	343.13	303.44	78.06
2679.29	67.14	267.33	268.88	61.83
2681.25	68.69	348.13	287.64	81.61
2683.20	93.34	282.79	254.91	104.37
2685.16	78.23	189.55	235.64	38.74
2687.11	71.78	136.26	218.77	69.05
2689.07	58.62	299.78	221.81	83.18
2691.02	92.97	288.63	222.94	94.60
2692.97	71.96	348.73	225.97	105.21
2694.92	49.15	365.59	216.01	112.53
2696.88	65.84	377.92	206.74	114.91
2698.83	68.48	305.94	208.41	146.93
2700.78	88.37	242.14	169.84	127.04
2702.73	76.61	167.38	162.27	124.51
2704.67	72.89	152.36	156.54	104.17
2706.62	79.48	298.72	163.60	119.39
2708.57	73.96	347.72	186.84	98.01
2710.51	98.84	236.27	146.24	79.01
2712.46	45.18	216.42	144.34	80.53
2714.40	42.21	218.14	140.73	60.88
2716.35	93.69	343.56	160.60	61.95
2718.29	83.17	281.00	140.50	117.40
2720.23	62.89	363.31	158.06	121.96
2722.18	64.42	220.80	117.60	121.37
2724.12	64.34	218.99	118.89	87.37
2726.06	68.12	333.07	122.69	90.87
2728.00	35.84	312.05	122.59	73.37
2729.94	62.26	356.29	130.89	77.02
2731.88	39.23	132.63	91.82	83.81
2733.81	75.50	259.66	100.22	88.90
2735.75	66.06	293.07	102.58	91.34
2737.69	91.03	203.45	89.68	113.18
2739.62	69.24	348.09	111.81	140.46
2741.56	68.60	277.28	97.41	118.19
2743.49	76.46	141.32	76.84	98.21
2745.42	22.57	339.37	128.10	99.88
2747.36	70.78	272.00	96.00	122.26
2749.29	51.83	306.44	91.80	79.93
2751.22	57.93	326.72	93.99	90.94

2753.15	60.49	272.32	82.82	113.70
2755.08	69.04	339.82	101.82	145.91
2757.01	84.48	129.13	62.41	104.36
2758.94	56.38	343.94	98.61	91.56
2760.86	58.67	331.17	93.24	146.90
2762.79	77.72	323.76	83.00	112.19
2764.72	58.16	175.90	60.76	147.08
2766.64	96.75	246.84	70.73	108.31
2768.57	66.43	315.67	118.65	117.20
2770.49	35.02	184.63	60.22	54.72
2772.41	71.00	170.16	63.34	121.13
2774.34	69.98	313.93	75.04	123.46
2776.26	75.66	305.87	85.43	113.07
2778.18	64.99	271.28	80.63	112.48
2780.10	62.81	76.50	41.15	116.24
2782.02	55.33	67.07	43.71	114.25
2783.94	87.56	270.94	90.91	72.45
2785.86	95.22	263.18	84.60	86.15
2787.77	40.44	244.63	64.03	67.59
2789.69	88.15	260.79	63.69	75.44
2791.61	72.36	118.18	46.98	79.39
2793.52	91.12	198.03	56.57	31.02
2795.44	94.58	29.61	35.40	110.67
2797.35	59.33	182.55	54.79	95.35
2799.26	73.24	109.34	43.44	122.83
2801.18	63.69	240.47	74.97	125.17
2803.09	85.59	102.85	45.23	102.50
2805.00	87.88	127.05	41.85	128.62
2806.91	79.62	99.84	44.31	126.55
2808.82	79.86	127.03	45.17	127.82
2810.73	94.75	39.90	36.39	140.70
2812.64	108.09	43.73	41.68	134.68
2814.54	69.82	160.95	56.43	139.69
2816.45	62.75	216.65	58.59	145.11
2818.36	88.68	175.01	58.28	104.69
2820.26	60.00	245.89	71.73	158.40
2822.17	139.77	172.18	56.86	153.33
2824.07	48.39	184.57	67.81	123.04
2825.97	106.31	280.67	89.73	211.05
2827.88	114.77	128.48	47.52	198.63
2829.78	125.04	85.16	47.94	195.54
2831.68	124.54	237.05	67.69	212.36
2833.58	89.00	243.01	98.68	156.38
2835.48	98.81	145.46	48.97	227.84

2837.38	94.71	300.39	95.12	218.91
2839.28	116.61	170.19	61.64	220.73
2841.17	122.80	240.00	72.36	168.59
2843.07	121.45	151.60	61.84	179.01
2844.97	80.94	166.24	63.50	207.99
2846.86	122.73	158.88	60.11	207.75
2848.76	95.71	280.34	75.40	198.08
2850.65	103.75	108.00	54.45	195.45
2852.54	90.48	213.87	67.60	201.21
2854.44	110.61	289.15	77.39	187.50
2856.33	85.54	230.92	103.94	209.92
2858.22	116.06	233.81	80.42	210.85
2860.11	142.63	276.67	93.73	189.27
2862.00	116.40	260.31	88.25	196.10
2863.89	109.02	198.55	74.00	192.39
2865.78	87.68	275.53	92.75	170.87
2867.67	110.95	265.56	95.47	146.71
2869.55	120.36	273.53	89.48	153.84
2871.44	94.76	77.63	65.43	164.38
2873.33	106.92	160.57	74.01	179.33
2875.21	138.77	255.63	95.99	163.37
2877.09	125.82	269.72	99.34	152.96
2878.98	83.77	193.88	85.29	149.27
2880.86	115.27	234.67	99.23	143.12
2882.74	102.76	304.39	108.91	183.47
2884.62	99.31	150.48	75.76	155.88
2886.50	114.35	264.59	127.67	173.68
2888.38	84.33	196.29	87.39	199.75
2890.26	93.27	204.64	101.66	148.57
2892.14	95.30	299.90	120.48	206.09
2894.02	101.28	219.67	104.32	162.76
2895.90	100.41	225.18	98.90	141.29
2897.77	113.09	211.55	100.95	177.87
2899.65	142.81	156.98	93.99	193.00
2901.53	112.99	288.97	117.70	207.55
2903.40	102.71	170.65	108.38	214.83
2905.27	129.23	167.73	101.62	176.08
2907.15	92.74	174.32	99.83	140.18
2909.02	124.21	263.07	118.18	147.78
2910.89	93.77	234.56	110.22	124.04
2912.76	140.98	120.55	96.40	133.40
2914.63	102.04	105.32	100.41	116.17
2916.50	99.59	315.46	117.81	150.99
2918.37	123.50	179.08	106.66	149.07

2920.24	116.95	224.35	114.83	120.00
2922.10	108.45	178.49	109.61	159.23
2923.97	90.65	234.98	130.32	125.57
2925.84	113.54	316.07	156.92	116.82
2927.70	129.79	193.89	116.13	119.41
2929.57	139.93	276.30	129.94	105.42
2931.43	156.37	275.11	135.28	96.02
2933.29	120.41	310.98	135.79	91.28
2935.16	89.44	255.41	147.76	98.35
2937.02	109.43	156.04	119.60	82.17
2938.88	117.06	216.31	125.41	103.03
2940.74	115.04	279.92	140.61	80.41
2942.60	123.62	108.24	119.08	94.93
2944.46	156.30	187.22	129.42	78.36
2946.32	172.42	253.46	136.46	93.99
2948.17	135.35	297.44	145.53	71.61
2950.03	183.57	248.89	144.24	110.30
2951.89	112.59	195.47	126.01	150.09
2953.74	157.01	254.18	131.68	134.87
2955.60	133.12	334.02	155.52	121.16
2957.45	165.19	247.87	136.69	109.90
2959.31	154.80	232.49	130.46	125.39
2961.16	155.76	181.02	131.36	130.28
2963.01	167.47	303.57	145.10	104.32
2964.86	162.53	333.53	156.04	119.61
2966.71	173.28	292.05	134.11	143.95
2968.56	162.94	237.61	125.28	109.64
2970.41	169.59	313.94	137.91	133.78
2972.26	151.79	329.77	139.78	95.67
2974.11	208.89	216.07	127.12	100.71
2975.96	204.09	365.71	150.05	87.70
2977.80	167.43	315.88	139.05	114.73
2979.65	178.17	218.55	121.92	67.47
2981.49	204.92	307.42	136.89	102.59
2983.34	194.96	319.35	151.82	68.68
2985.18	129.90	236.07	126.02	35.16
2987.02	201.68	255.06	137.46	70.53
2988.87	208.27	280.70	136.62	53.67
2990.71	192.55	358.08	164.59	46.44
2992.55	158.09	325.71	155.06	49.10
2994.39	221.32	263.45	141.62	46.57
2996.23	182.00	287.85	142.89	70.84
2998.07	197.43	262.84	142.59	67.25
2999.91	214.35	140.33	130.15	49.81

3001.74	217.03	274.54	148.28	52.00
3003.58	225.25	284.39	158.02	73.45
3005.42	202.67	287.76	164.28	54.96
3007.25	230.34	149.78	141.68	98.69
3009.09	254.81	135.46	139.21	58.63
3010.92	233.53	336.91	171.07	61.21
3012.75	222.30	208.19	158.24	78.13
3014.59	267.11	272.17	173.84	93.62
3016.42	295.77	289.74	167.80	112.53
3018.25	262.39	213.80	164.60	111.54
3020.08	322.15	284.53	168.73	112.40
3021.91	282.46	221.51	166.42	137.50
3023.74	285.36	323.18	187.12	117.04
3025.57	266.07	293.26	183.28	114.03
3027.40	298.08	355.17	204.44	143.67
3029.22	293.48	291.18	196.94	173.94
3031.05	300.73	391.14	219.80	175.21
3032.88	319.28	167.36	178.56	141.38
3034.70	325.68	229.68	194.79	135.78
3036.53	308.28	354.34	220.05	155.63
3038.35	357.53	291.54	216.28	109.77
3040.17	327.27	288.40	214.21	129.20
3041.99	351.97	290.18	216.93	122.07
3043.82	383.91	311.86	238.99	115.30
3045.64	382.30	365.17	238.55	118.66
3047.46	403.49	273.38	234.55	106.31
3049.28	369.08	370.43	254.91	109.06
3051.10	382.97	348.00	256.00	97.89
3052.91	416.31	358.07	269.36	115.71
3054.73	416.34	137.08	236.98	109.23
3056.55	415.58	158.47	234.64	116.19
3058.37	427.06	333.70	302.20	116.34
3060.18	421.99	305.66	273.66	110.03
3062.00	447.33	364.62	290.32	148.26
3063.81	432.05	282.50	283.94	121.58
3065.62	462.13	188.46	274.60	158.33
3067.44	468.01	204.23	285.06	156.54
3069.25	484.99	234.11	286.25	113.13
3071.06	500.66	222.83	290.24	106.40
3072.87	475.04	211.91	303.17	132.47
3074.68	498.11	330.61	345.12	108.98
3076.49	492.23	233.81	320.28	102.33
3078.30	508.60	352.31	347.80	121.61
3080.11	557.02	173.72	328.16	122.34

3081.91	551.44	383.97	370.88	131.04
3083.72	541.76	267.08	346.20	119.35
3085.53	562.38	411.79	374.63	127.24
3087.33	600.14	329.13	375.25	89.31
3089.14	616.66	269.13	370.87	89.03
3090.94	619.52	212.76	379.69	106.58
3092.74	608.78	422.04	412.95	131.52
3094.55	639.54	397.92	420.20	83.75
3096.35	623.60	434.51	433.02	100.71
3098.15	654.76	373.20	443.21	135.56
3099.95	680.72	292.90	434.03	157.56
3101.75	646.93	430.30	467.12	138.24
3103.55	707.44	400.59	489.51	146.81
3105.35	732.04	277.69	463.96	104.07
3107.15	728.10	326.41	481.11	101.17
3108.94	755.10	370.04	513.47	113.11
3110.74	725.86	370.31	515.79	44.10
3112.53	778.71	331.25	522.54	68.17
3114.33	785.06	333.26	544.86	37.39
3116.12	821.01	369.91	558.71	45.60
3117.92	818.36	372.76	564.30	56.66
3119.71	819.26	368.75	596.65	74.96
3121.50	883.91	370.12	607.13	72.50
3123.29	881.75	253.59	591.42	58.44
3125.09	913.10	183.85	593.04	66.69
3126.88	928.24	424.34	652.35	89.87
3128.67	960.79	234.05	628.64	102.75
3130.46	980.13	242.14	641.39	65.33
3132.24	981.23	364.45	678.61	52.05
3134.03	1014.17	283.36	681.26	49.07
3135.82	1032.76	457.29	719.14	55.30
3137.60	1036.60	367.29	717.36	70.86
3139.39	1080.59	428.36	732.87	40.19
3141.17	1096.13	516.15	772.62	7.06
3142.96	1105.17	505.25	779.14	32.19
3144.74	1189.85	480.99	786.29	27.86
3146.53	1180.29	621.32	847.31	55.19
3148.31	1217.88	628.17	839.49	86.12
3150.09	1252.01	604.11	837.80	85.15
3151.87	1283.15	562.18	847.39	96.73
3153.65	1322.78	844.15	892.63	96.97
3155.43	1386.27	932.65	916.95	104.41
3157.21	1395.85	955.89	942.50	77.31
3158.99	1430.53	1050.86	942.41	70.72

3160.76	1456.11	1126.75	958.66	48.27
3162.54	1493.64	1163.63	966.44	82.84
3164.32	1547.22	1312.28	976.55	88.96
3166.09	1560.10	1463.13	1001.30	87.68
3167.87	1608.98	1572.30	1018.78	81.08
3169.64	1642.46	1698.49	1022.06	63.67
3171.42	1686.19	1943.81	1070.68	68.03
3173.19	1775.01	2104.03	1067.32	102.79
3174.96	1778.74	2121.51	1081.77	85.56
3176.73	1798.97	2140.49	1092.05	59.64
3178.50	1860.69	2329.84	1120.23	60.58
3180.27	1898.62	2300.32	1135.64	69.08
3182.04	1953.19	2127.42	1121.32	90.55
3183.81	1984.86	2249.98	1155.03	62.78
3185.58	2010.44	2272.50	1187.51	19.82
3187.35	2113.21	2121.89	1176.29	31.27
3189.11	2171.48	2126.78	1203.37	30.53
3190.88	2185.65	2043.03	1211.18	34.66
3192.65	2236.78	2122.46	1233.86	29.11
3194.41	2281.10	2104.00	1261.00	38.17
3196.17	2334.72	2021.51	1249.21	51.39
3197.94	2369.34	2111.23	1279.99	56.08
3199.70	2430.56	2291.45	1323.70	54.03
3201.46	2456.72	2303.77	1323.58	50.70
3203.22	2546.04	2397.02	1346.49	96.84
3204.99	2557.01	2581.59	1365.33	57.36
3206.75	2636.58	2893.99	1405.81	44.73
3208.50	2668.50	2969.06	1418.02	66.11
3210.26	2681.91	3252.49	1448.96	36.23
3212.02	2802.28	3640.82	1510.77	92.96
3213.78	2819.84	3822.36	1503.68	94.87
3215.54	2880.81	4289.92	1562.55	112.99
3217.29	2978.08	4614.34	1593.96	94.17
3219.05	3029.44	4922.81	1616.47	99.04
3220.80	3090.55	5163.23	1643.51	95.17
3222.56	3177.52	5399.84	1679.42	110.79
3224.31	3228.63	5498.53	1712.20	91.26
3226.06	3279.05	5656.47	1760.17	104.26
3227.81	3353.51	5591.06	1789.54	88.63
3229.57	3426.17	5585.26	1845.19	112.79
3231.32	3481.08	5491.92	1910.09	113.41
3233.07	3515.10	5263.53	1943.03	146.91
3234.82	3631.56	4931.82	1975.11	131.23
3236.57	3712.17	4767.53	2025.68	149.13

3238.31	3744.88	4567.79	2076.19	146.99
3240.06	3887.49	4402.81	2134.46	106.65
3241.81	3919.10	4168.93	2155.13	120.28
3243.55	3965.81	4029.91	2188.84	127.08
3245.30	4080.02	3887.70	2220.55	143.07
3247.04	4137.93	4006.87	2298.89	126.27
3248.79	4224.09	3910.77	2349.19	118.02
3250.53	4320.35	3978.09	2374.96	149.33
3252.27	4441.41	3762.91	2351.90	113.62
3254.02	4523.17	3835.93	2390.88	128.41
3255.76	4620.78	3874.10	2424.15	124.07
3257.50	4709.49	4010.13	2444.45	168.15
3259.24	4788.35	4076.41	2467.46	143.02
3260.98	4827.91	4281.06	2492.69	146.53
3262.72	4971.06	4578.09	2497.07	116.61
3264.46	5054.87	4810.46	2516.87	118.03
3266.19	5122.78	5384.13	2545.57	116.51
3267.93	5215.09	5715.45	2517.88	110.45
3269.67	5244.20	6248.06	2531.88	104.67
3271.40	5347.50	6714.82	2526.05	98.61
3273.14	5325.61	7274.10	2539.59	106.90
3274.87	5412.32	8008.57	2586.09	87.37
3276.60	5428.32	8467.42	2572.40	88.51
3278.34	5426.83	8720.66	2553.73	91.52
3280.07	5472.49	9013.94	2549.20	99.24
3281.80	5467.99	9353.79	2567.91	101.70
3283.53	5506.80	9443.53	2577.44	105.34
3285.26	5412.31	9395.46	2582.94	100.58
3286.99	5434.32	9223.63	2588.98	107.15
3288.72	5369.97	9006.30	2606.72	78.72
3290.45	5371.38	8511.03	2615.08	100.17
3292.18	5350.99	7884.09	2607.62	95.57
3293.90	5330.49	7397.66	2623.29	67.73
3295.63	5319.75	6665.35	2611.96	45.01
3297.35	5263.40	6252.67	2618.06	73.41
3299.08	5274.86	5856.68	2647.92	95.22
3300.80	5234.07	5559.83	2679.49	120.70
3302.53	5250.62	5085.66	2636.16	85.99
3304.25	5201.63	4870.44	2654.53	50.91
3305.97	5169.39	4850.48	2695.83	81.37
3307.69	5189.29	4536.69	2679.43	54.38
3309.42	5155.05	4552.67	2723.83	120.00
3311.14	5106.36	4487.95	2720.10	84.49
3312.86	5133.96	4408.29	2730.93	96.58

3314.57	5122.32	4664.69	2789.06	51.24
3316.29	5138.53	4642.74	2789.60	63.62
3318.01	5128.98	4703.60	2806.86	61.29
3319.73	5122.59	4847.88	2858.06	47.35
3321.44	5175.85	4825.75	2847.66	62.99
3323.16	5137.65	4867.42	2860.89	101.29
3324.87	5212.11	5021.43	2899.76	104.25
3326.59	5202.47	5180.06	2917.79	82.54
3328.30	5203.18	5276.26	2942.82	59.09
3330.02	5255.68	5353.00	2977.96	106.51
3331.73	5231.59	5369.95	2974.65	62.94
3333.44	5303.55	5538.89	3023.89	81.06
3335.15	5327.11	5491.08	3041.65	93.28
3336.86	5321.32	5561.84	3076.11	74.83
3338.57	5390.77	5663.22	3109.91	94.07
3340.28	5394.13	5701.26	3115.51	22.01
3341.99	5395.99	5686.83	3150.74	30.38
3343.70	5426.85	5701.86	3171.37	71.49
3345.41	5507.81	5693.90	3166.50	57.59
3347.11	5534.02	5603.73	3154.13	73.31
3348.82	5526.63	5868.56	3197.43	79.23
3350.52	5636.59	5870.66	3208.36	71.87
3352.23	5653.15	6008.92	3238.46	79.27
3353.93	5667.11	6008.41	3225.35	63.63
3355.63	5754.47	6105.40	3234.08	78.43
3357.34	5772.53	6118.47	3243.61	85.39
3359.04	5838.44	6124.21	3248.71	82.46
3360.74	5845.90	6260.12	3280.90	99.72
3362.44	5940.36	6409.76	3318.73	55.81
3364.14	5977.42	6526.23	3341.93	82.48
3365.84	6018.38	6477.15	3342.99	82.25
3367.54	6050.00	6573.32	3377.92	82.64
3369.24	6125.96	6768.79	3437.68	113.17
3370.94	6182.92	6724.64	3428.08	84.24
3372.63	6221.39	7059.99	3493.31	101.30
3374.33	6273.20	7185.34	3534.00	49.39
3376.02	6316.61	7322.12	3559.79	76.97
3377.72	6427.48	7703.95	3626.89	115.47
3379.41	6510.69	7842.61	3648.12	97.87
3381.11	6469.06	8251.75	3730.98	54.38
3382.80	6650.57	8454.63	3730.07	106.09
3384.49	6638.83	8814.37	3787.37	57.49
3386.18	6699.85	9093.20	3848.26	71.71
3387.87	6772.32	9167.81	3875.65	90.12

3389.56	6809.78	9382.76	3946.01	74.71
3391.25	6881.35	9352.28	3971.31	117.08
3392.94	6902.07	9092.90	3950.63	142.92
3394.63	6976.23	9156.38	4001.23	128.40
3396.32	7017.60	9104.19	4042.02	138.86
3398.01	7038.42	8767.29	4040.55	143.15
3399.69	7037.59	8713.82	4066.24	121.33
3401.38	7050.26	8529.28	4063.50	121.33
3403.06	7058.33	8511.00	4094.12	91.07
3404.75	7020.00	8338.04	4096.82	99.57
3406.43	7034.42	8217.68	4110.44	94.06
3408.12	7031.84	8094.16	4106.77	100.27
3409.80	6953.26	8114.34	4128.06	124.22
3411.48	6995.08	7877.78	4102.72	125.85
3413.16	6954.75	8013.39	4120.44	108.80
3414.84	6891.68	8076.54	4119.34	104.78
3416.52	6858.70	8225.35	4133.79	98.44
3418.20	6828.52	8415.29	4115.05	65.37
3419.88	6728.35	8464.09	4098.11	68.38
3421.56	6729.52	8681.74	4117.00	105.36
3423.24	6641.30	8620.53	4070.56	98.37
3424.91	6610.07	8816.54	4076.32	47.27
3426.59	6535.55	8788.56	4050.41	86.53
3428.26	6471.62	8696.69	3989.96	77.27
3429.94	6427.60	8535.66	3959.29	78.48
3431.61	6335.38	8508.14	3931.28	74.37
3433.29	6293.56	8215.62	3859.90	97.72
3434.96	6227.14	7980.41	3828.96	129.96
3436.63	6155.91	7567.29	3747.38	132.82
3438.30	6085.59	7385.75	3687.97	172.21
3439.97	6027.52	7148.70	3647.06	154.66
3441.64	5893.31	6911.35	3591.82	134.44
3443.31	5844.14	6619.58	3515.31	158.24
3444.98	5762.77	6345.46	3442.10	165.56
3446.65	5691.90	5919.60	3371.62	170.40
3448.32	5572.48	5696.15	3304.17	125.22
3449.99	5469.02	5359.96	3229.86	123.95
3451.65	5408.80	5207.03	3175.12	109.67
3453.32	5256.49	4927.15	3107.91	123.29
3454.98	5242.17	4795.26	3057.23	141.09
3456.65	5102.66	4428.41	2961.25	148.81
3458.31	5008.00	4371.34	2923.37	135.65
3459.97	4973.88	4214.61	2883.59	126.86
3461.64	4837.92	4003.06	2812.35	171.29

3463.30	4746.61	4012.52	2768.54	151.78
3464.96	4675.30	3807.68	2704.26	139.15
3466.62	4539.24	3700.09	2652.95	148.29
3468.28	4453.48	3625.00	2593.43	154.89
3469.94	4339.07	3461.89	2533.09	154.91
3471.60	4282.71	3443.51	2515.21	111.30
3473.26	4208.76	3288.03	2448.40	147.85
3474.91	4124.95	3209.61	2411.18	135.83
3476.57	4061.79	3200.28	2393.47	133.42
3478.23	3955.89	3044.80	2342.69	134.07
3479.88	3908.93	2931.71	2300.18	152.55
3481.54	3764.88	2679.71	2243.00	129.29
3483.19	3719.83	2617.29	2216.45	122.89
3484.85	3644.82	2568.40	2192.20	159.76
3486.50	3547.27	2488.37	2156.86	119.85
3488.15	3479.82	2277.23	2108.94	156.90
3489.80	3388.02	2163.52	2086.06	120.97
3491.45	3377.02	2129.49	2056.48	122.25
3493.10	3292.22	2082.61	2040.20	152.19
3494.75	3159.07	2049.62	2022.22	136.80
3496.40	3123.88	1938.64	1984.84	120.11
3498.05	3038.58	1734.29	1945.39	88.89
3499.70	2940.44	1763.75	1935.97	112.79
3501.35	2875.44	1681.14	1910.93	111.39
3502.99	2793.80	1575.95	1879.81	112.46
3504.64	2677.60	1625.10	1898.06	88.69
3506.28	2673.61	1545.27	1857.15	125.23
3507.93	2580.37	1502.29	1851.17	114.72
3509.57	2548.88	1562.08	1845.58	120.48
3511.22	2463.94	1314.06	1804.17	99.64
3512.86	2380.95	1471.13	1835.02	105.36
3514.50	2348.51	1373.21	1803.37	105.04
3516.14	2285.82	1393.55	1807.72	93.98
3517.78	2189.58	1162.15	1766.24	107.92
3519.42	2208.60	1343.49	1794.59	128.12
3521.06	2108.76	1162.56	1774.24	117.73
3522.70	2091.98	1178.20	1786.19	163.54
3524.34	2004.84	1150.23	1768.87	124.85
3525.98	1996.06	974.03	1766.59	144.37
3527.62	1933.23	1028.48	1758.11	141.79
3529.25	1866.45	999.99	1752.79	135.38
3530.89	1804.42	1065.36	1769.91	144.20
3532.52	1782.94	1010.07	1753.42	162.14
3534.16	1688.76	1047.23	1736.67	157.07

3535.79	1687.53	911.32	1715.26	144.96
3537.43	1609.66	916.08	1684.30	159.45
3539.06	1578.63	937.81	1678.49	161.49
3540.69	1529.81	910.07	1656.14	146.93
3542.32	1479.08	763.31	1588.65	163.57
3543.95	1466.41	714.57	1566.70	151.76
3545.58	1387.39	714.65	1540.38	137.60
3547.21	1377.82	743.02	1516.36	107.18
3548.84	1356.35	613.20	1468.65	122.51
3550.47	1291.73	604.49	1434.06	113.24
3552.10	1288.11	559.59	1412.18	130.72
3553.72	1251.39	636.86	1388.66	55.30
3555.35	1193.42	632.43	1356.77	65.82
3556.98	1166.16	502.06	1320.19	93.14
3558.60	1145.34	529.83	1295.00	64.45
3560.22	1109.03	433.37	1250.35	74.71
3561.85	1087.42	344.28	1224.30	69.01
3563.47	1057.91	514.74	1249.38	94.41
3565.09	1031.54	477.40	1201.29	137.00
3566.72	992.84	486.57	1187.81	129.43
3568.34	985.18	399.79	1127.46	112.31
3569.96	968.77	359.11	1108.54	101.93
3571.58	962.66	497.61	1114.68	86.99
3573.20	922.06	437.04	1066.56	105.34
3574.82	860.30	494.44	1055.18	134.28
3576.43	871.50	499.54	1029.99	137.32
3578.05	857.14	441.06	1001.20	172.09
3579.67	846.54	308.60	969.98	158.10
3581.28	805.14	318.51	934.93	199.51
3582.90	830.99	382.60	935.28	169.25
3584.52	785.64	328.34	901.92	149.82
3586.13	774.35	506.14	905.47	155.00
3587.74	743.25	452.60	876.91	201.61
3589.36	746.76	441.91	860.06	148.39
3590.97	722.21	490.48	846.07	168.27
3592.58	671.52	373.06	810.62	162.19
3594.19	705.12	472.90	787.96	153.65
3595.80	681.78	381.48	757.68	152.58
3597.41	639.39	421.75	750.75	157.31
3599.02	646.45	486.52	756.73	143.56
3600.63	594.77	455.19	712.78	124.45
3602.24	599.73	393.71	689.99	134.03
3603.85	571.54	325.45	661.20	173.49
3605.45	541.11	203.92	621.68	156.58

3607.06	535.73	294.62	619.69	150.56
3608.66	488.19	377.88	625.24	181.92
3610.27	507.61	331.20	593.85	158.40
3611.87	517.78	313.30	574.33	137.28
3613.48	478.20	376.86	581.97	127.67
3615.08	501.57	178.83	527.21	118.71
3616.68	466.10	370.81	530.86	164.82
3618.29	444.37	334.06	514.97	171.26
3619.89	416.30	231.32	482.95	169.93
3621.49	440.42	183.40	455.12	131.73
3623.09	419.35	265.05	459.94	182.11
3624.69	411.03	253.52	441.78	185.46
3626.28	395.11	366.32	450.59	182.06
3627.88	397.09	387.94	430.17	172.37
3629.48	388.97	392.98	426.08	179.56
3631.08	385.31	412.03	413.75	173.42
3632.67	384.39	339.30	391.10	184.22
3634.27	339.28	226.59	356.57	172.03
3635.86	328.76	365.00	363.25	154.68
3637.46	344.55	294.52	347.13	166.69
3639.05	349.49	203.90	321.40	160.79
3640.64	321.23	303.76	319.11	150.91
3642.24	310.23	391.25	326.42	146.89
3643.83	304.87	344.39	311.30	147.66
3645.42	311.56	399.09	312.24	150.55
3647.01	264.16	183.00	274.35	111.91
3648.60	301.91	211.06	265.52	122.84
3650.19	269.70	354.68	280.37	148.30
3651.78	219.25	373.16	276.67	124.32
3653.37	256.30	370.79	263.38	149.37
3654.95	219.85	348.19	256.16	136.70
3656.54	241.11	199.53	223.27	144.53
3658.13	217.86	196.24	225.21	150.23
3659.71	240.27	305.39	233.08	139.07
3661.30	210.17	378.59	227.49	165.01
3662.88	190.83	370.78	237.63	141.23
3664.47	159.94	311.76	209.44	118.41
3666.05	123.00	321.08	203.88	102.82
3667.63	208.16	226.11	197.56	119.64
3669.21	165.78	389.91	211.70	153.19
3670.80	182.24	183.86	173.47	116.79
3672.38	164.16	149.88	166.08	119.92
3673.96	154.32	232.19	164.99	106.22
3675.54	181.24	323.54	172.83	109.69

3677.11	141.36	355.51	181.87	98.01
3678.69	148.98	355.13	169.68	146.42
3680.27	148.15	213.80	150.22	125.06
3681.85	107.08	369.18	175.06	110.62
3683.42	146.10	355.05	167.53	123.67
3685.00	134.78	401.70	163.54	151.33
3686.57	130.05	260.71	142.21	135.63
3688.15	148.28	327.81	149.35	132.88
3689.72	130.56	389.50	157.02	116.53
3691.30	177.05	289.67	132.43	136.24
3692.87	103.18	285.95	133.57	134.44
3694.44	122.76	396.65	147.64	171.42
3696.01	123.50	227.73	112.42	168.18
3697.58	130.23	266.40	114.19	163.01
3699.15	110.52	324.43	112.66	141.32
3700.72	110.21	320.79	113.50	156.01
3702.29	145.30	293.55	109.97	157.78
3703.86	81.04	318.79	106.78	172.38
3705.43	118.14	369.38	116.65	134.21
3706.99	105.23	377.14	112.69	134.64
3708.56	106.23	399.83	122.50	147.43
3710.13	88.63	363.58	110.00	147.26
3711.69	84.17	364.92	109.48	126.68
3713.26	98.38	350.49	100.75	100.77
3714.82	109.33	313.42	97.42	141.87
3716.38	102.53	363.38	105.09	117.34
3717.95	114.49	376.29	102.00	106.33
3719.51	78.64	418.21	138.40	103.53
3721.07	80.50	213.64	81.87	119.81
3722.63	99.36	356.33	100.51	108.93
3724.19	78.67	302.05	89.78	157.59
3725.75	81.98	425.95	99.65	125.53
3727.31	75.44	349.26	94.89	137.91
3728.87	80.31	206.30	66.80	157.72
3730.43	64.32	330.10	87.50	136.93
3731.98	103.14	257.46	81.24	117.64
3733.54	68.01	196.42	68.31	107.18
3735.10	69.03	182.89	61.28	80.90
3736.65	89.30	391.59	118.62	86.93
3738.21	49.03	319.62	87.12	81.24
3739.76	62.50	313.86	86.59	75.06
3741.31	53.77	193.17	62.00	93.12
3742.87	75.40	294.63	80.57	90.55
3744.42	66.23	307.34	76.50	109.74

3745.97	94.71	244.24	70.61	100.83
3747.52	87.74	329.73	80.41	119.71
3749.07	55.18	359.30	91.35	121.19
3750.62	83.51	229.21	63.35	92.41
3752.17	85.95	316.95	77.12	114.13
3753.72	62.44	252.00	65.69	127.69
3755.27	55.43	330.66	72.03	124.50
3756.82	52.32	264.06	69.46	105.55
3758.36	73.11	273.79	61.87	169.98
3759.91	66.40	399.78	88.64	96.93
3761.45	54.45	298.12	66.31	143.30
3763.00	86.49	206.84	53.11	131.09
3764.54	65.34	377.84	70.48	137.91
3766.09	65.49	279.48	63.28	153.16
3767.63	73.84	345.19	67.02	144.62
3769.17	47.45	294.05	59.48	139.54
3770.72	71.25	278.17	61.59	135.14
3772.26	84.71	238.09	48.66	136.46
3773.80	119.92	450.66	78.86	155.38
3775.34	64.12	368.03	61.33	189.01
3776.88	92.98	315.97	65.10	148.73
3778.42	83.20	363.76	65.53	141.67
3779.95	91.31	415.11	74.93	120.52
3781.49	72.87	278.36	48.70	146.56
3783.03	55.29	314.18	52.70	172.61
3784.57	100.71	213.19	44.57	133.51
3786.10	74.08	324.15	60.27	118.53
3787.64	72.30	266.15	54.68	159.99
3789.17	63.12	331.45	61.11	171.83
3790.70	63.39	247.85	49.94	144.08
3792.24	67.62	371.67	73.45	151.91
3793.77	32.99	230.31	51.25	144.82
3795.30	37.77	290.68	55.28	131.69
3796.84	114.10	223.08	40.28	126.10
3798.37	27.63	289.07	58.65	158.94
3799.90	46.46	152.47	46.98	138.34
3801.43	45.89	204.29	47.55	151.50
3802.96	81.27	198.58	47.89	113.02
3804.48	77.01	121.19	32.45	114.27
3806.01	40.44	343.88	64.25	121.06
3807.54	93.88	257.47	57.72	118.42
3809.07	61.32	278.46	58.46	116.38
3810.59	66.11	196.11	49.59	96.88
3812.12	12.65	267.28	56.22	152.22

3813.64	77.79	120.37	33.86	113.45
3815.17	81.63	210.25	49.52	111.81
3816.69	64.42	194.79	40.59	123.26
3818.21	83.27	199.72	45.69	143.02
3819.74	45.67	263.49	45.86	144.37
3821.26	52.56	260.63	47.72	161.86
3822.78	53.86	306.29	70.89	144.65
3824.30	41.11	303.91	60.99	146.86
3825.82	51.56	208.72	34.19	147.38
3827.34	101.76	205.92	44.16	123.81
3828.86	65.96	298.65	55.29	145.55
3830.38	55.61	349.27	72.52	143.25
3831.89	80.66	347.51	70.82	172.48
3833.41	46.27	375.16	60.89	162.60
3834.93	55.08	235.32	49.19	142.18
3836.44	93.58	157.94	41.78	145.05
3837.96	51.84	359.41	83.02	142.30
3839.47	99.60	190.44	35.68	144.09
3840.99	76.81	303.98	53.85	152.41
3842.50	41.57	381.91	63.21	165.47
3844.01	82.73	370.10	63.65	154.48
3845.53	64.14	242.20	46.91	144.49
3847.04	90.40	242.64	49.64	166.97
3848.55	86.72	333.43	58.88	156.52
3850.06	57.88	362.29	59.54	128.37
3851.57	60.14	265.88	46.24	143.43
3853.08	67.46	280.60	41.67	160.41
3854.59	119.88	250.77	39.60	139.84
3856.10	69.79	276.89	47.70	137.63
3857.60	34.41	257.68	55.70	139.20
3859.11	66.58	391.45	72.60	144.71
3860.62	91.30	401.47	73.16	122.73
3862.12	97.87	351.14	68.66	118.37
3863.63	52.29	407.87	65.46	85.71
3865.13	53.71	351.53	57.79	98.57
3866.64	98.63	324.99	49.69	107.98
3868.14	56.45	368.44	67.09	108.31
3869.64	79.67	331.13	50.42	141.70
3871.14	70.60	223.83	30.35	140.19
3872.65	69.42	384.94	86.25	137.18
3874.15	49.85	362.16	68.28	142.39
3875.65	61.17	368.60	58.91	132.52
3877.15	79.50	284.21	48.60	131.84
3878.65	39.27	347.86	90.74	126.33

3880.14	89.35	375.55	58.97	136.59
3881.64	55.82	302.42	54.66	97.14
3883.14	68.10	233.07	32.89	131.50
3884.64	33.38	307.20	52.62	114.77
3886.13	51.61	174.62	37.05	121.96
3887.63	59.09	176.20	25.75	95.19
3889.12	63.87	160.50	30.58	92.50
3890.62	92.45	330.28	59.61	116.24
3892.11	76.13	217.62	42.91	102.11
3893.60	63.21	152.56	23.47	127.54
3895.10	70.44	133.90	31.87	102.30
3896.59	47.92	273.70	49.70	130.24
3898.08	34.40	294.65	44.46	103.34
3899.57	49.73	276.43	49.16	129.04
3901.06	83.61	213.37	46.12	152.25
3902.55	53.90	290.75	59.82	141.66
3904.04	28.53	303.53	51.48	118.93
3905.53	78.31	160.23	28.38	138.41
3907.01	44.24	341.65	72.74	161.81
3908.50	43.73	303.40	49.17	104.55
3909.99	57.76	268.88	49.27	152.04
3911.47	56.75	278.97	45.26	136.86
3912.96	66.03	242.31	52.06	150.21
3914.44	41.81	295.98	51.99	166.24
3915.93	62.80	177.51	35.22	162.01
3917.41	62.13	230.69	46.81	127.55
3918.89	83.32	256.85	44.97	172.69
3920.38	37.71	317.44	54.17	116.62
3921.86	54.39	335.00	62.90	129.77
3923.34	96.48	371.55	74.63	110.38
3924.82	76.61	277.61	55.59	112.69
3926.30	45.75	327.85	76.88	121.56
3927.78	68.88	267.77	52.75	99.88
3929.26	58.07	342.60	85.48	138.31
3930.74	56.81	232.74	55.37	111.84
3932.21	76.69	200.04	40.73	101.59
3933.69	49.43	251.05	76.23	99.08
3935.17	55.01	148.98	43.29	91.27
3936.64	35.40	150.16	43.75	124.24
3938.12	39.58	168.85	54.51	107.04
3939.59	35.62	190.20	52.34	90.71
3941.07	49.51	140.14	49.37	93.72
3942.54	26.49	30.74	35.80	122.80
3944.01	42.58	72.15	33.26	126.38

3945.49	74.16	107.21	48.39	115.05
3946.96	67.60	210.24	70.28	95.99
3948.43	43.53	41.53	28.41	121.78
3949.90	65.77	11.36	34.77	129.03
3951.37	61.86	168.77	77.23	126.82
3952.84	27.94	9.44	37.19	102.78
3954.31	54.18	207.40	69.89	115.43
3955.78	52.71	139.04	50.88	104.09
3957.24	48.85	250.43	101.24	89.51
3958.71	91.18	111.43	44.23	86.73
3960.18	80.32	182.55	54.96	149.74
3961.64	120.15	134.71	50.09	101.38
3963.11	72.38	178.64	55.45	132.10
3964.57	30.72	184.71	59.48	131.34
3966.04	68.50	119.88	53.51	133.46
3967.50	55.79	196.34	66.10	135.15
3968.96	39.77	169.56	68.06	167.80
3970.42	75.30	186.62	62.52	162.14
3971.89	73.19	178.55	61.71	112.23
3973.35	83.77	220.78	73.14	135.33
3974.81	63.95	122.10	50.57	150.38
3976.27	103.68	191.50	54.39	153.98
3977.73	52.06	123.67	56.95	163.64
3979.19	48.35	261.90	76.41	178.45
3980.64	100.17	208.31	65.24	163.82
3982.10	63.96	261.17	75.73	156.18
3983.56	80.19	272.96	95.26	143.99
3985.01	82.16	298.10	77.39	177.22
3986.47	82.59	99.53	51.98	181.03
3987.93	88.33	98.38	48.87	131.99
3989.38	101.65	177.75	56.57	150.77
3990.83	85.43	264.95	68.76	156.95
3992.29	91.16	335.71	90.55	133.87
3993.74	88.69	175.48	59.11	125.65
3995.19	91.21	248.73	60.60	196.53
3996.64	54.29	393.16	85.33	153.12
3998.10	69.47	319.53	64.86	154.08
3999.55	58.59	212.35	55.12	131.12
4001.00	111.67	392.69	85.71	140.60
4002.44	111.64	240.00	50.00	120.00

End of Supplementary Information
